# A T‐cell reporter platform for high‐throughput and reliable investigation of TCR function and biology

**DOI:** 10.1002/cti2.1216

**Published:** 2020-11-23

**Authors:** Thomas R Müller, Corinna Schuler, Monika Hammel, Amelie Köhler, Sabrina Jutz, Judith Leitner, Kilian Schober, Dirk H Busch, Peter Steinberger

**Affiliations:** ^1^ Institute for Medical Microbiology, Immunology and Hygiene Technical University of Munich (TUM) Munich Germany; ^2^ German Center for Infection Research (DZIF) Munich Germany; ^3^ Division of Immune Receptors and T Cell Activation Center for Pathophysiology, Infectiology, and Immunology Institute of Immunology Medical University of Vienna Vienna Austria; ^4^ Focus Group ‘Clinical Cell Processing and Purification’ Institute for Advanced Study TUM Munich Germany

**Keywords:** adoptive T‐cell therapy, Cas9, CRISPR, reporter T‐cell line, TCR biology, TCR functional avidity, TCR gene editing

## Abstract

**Objective:**

Transgenic re‐expression enables unbiased investigation of T‐cell receptor (TCR)‐intrinsic characteristics detached from its original cellular context. Recent advancements in TCR repertoire sequencing and development of protocols for direct cloning of full TCRαβ constructs now facilitate large‐scale transgenic TCR re‐expression. Together, this offers unprecedented opportunities for the screening of TCRs for basic research as well as clinical use. However, the functional characterisation of re‐expressed TCRs is still a complicated and laborious matter. Here, we propose a Jurkat‐based triple parameter TCR signalling reporter endogenous TCR knockout cellular platform (TPR^KO^) that offers an unbiased, easy read‐out of TCR functionality and facilitates high‐throughput screening approaches.

**Methods:**

As a proof‐of‐concept, we transgenically re‐expressed 59 human cytomegalovirus‐specific TCRs and systematically investigated and compared TCR function in TPR^KO^ cells versus primary human T cells.

**Results:**

We demonstrate that the TPR^KO^ cell line facilitates antigen‐HLA specificity screening via sensitive peptide‐MHC‐multimer staining, which was highly comparable to primary T cells. Also, TCR functional avidity in TPR^KO^ cells was strongly correlating to primary T cells, especially in the absence of CD8αβ co‐receptor.

**Conclusion:**

Overall, our data show that the TPR^KO^ cell lines can serve as a surrogate of primary human T cells for standardised and high‐throughput investigation of TCR biology.

## Graphical abstract

A highly sensitive triple parameter T‐cell receptor (TCR) reporter cell line was adapted for high‐throughput testing of transgenically expressed TCRs by CRISPR/Cas9‐mediated deletion of the endogenous TCR and introduction of the CD8αβ co‐receptor. Using 59 CMV‐specific TCRs, we demonstrated a high similarity with primary T cells in terms of multimer staining, functional avidity and CD8‐dependence. In conclusion, we present a T‐cell reporter platform excellently suited for high‐throughput functional preclinical testing of TCRs.

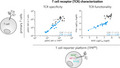

## Introduction

The genetic replacement of TCRs^1^, ^2^ facilitates reprogramming of a T cell’s antigen‐HLA specificity and offers exciting new prospects for basic research as well as adoptive cell therapy.^3^, ^4^ However, especially the identification and in‐depth characterisation of suitable TCRs for clinical use was so far a tedious process and only a handful of clinical studies with TCR re‐directed T‐cell products are reported.^5^, ^6^, ^7^, ^8^, ^9^ Today, because of continuous improvements in the field of next‐generation sequencing, high‐throughput identification of full αβ TCR sequences is no longer a bottleneck.^10^, ^11^, ^12^, ^13^ Moreover, advanced bioinformatical analytical tools are developed to gain deep insight into such large TCR repertoire data and to predict antigen‐HLA specificity from raw TCR sequences.^14^, ^15^ However, a major remaining hurdle is the functional testing of TCR candidates. Earlier studies characterised TCRs by *in vitro* generation and functional testing of T‐cell clones.^16^, ^17^, ^18^ Importantly, TCR function is affected by its cellular context, so that – for instance – the phenotype of a T‐cell clone affects TCR functional avidity or even specificity, as previously demonstrated with tumor‐infiltrating lymphocytes.^19^ Hence, transgenic re‐expression of TCRs in a suitable cell line or primary T cells^20^ is the most standardised approach to assess TCR‐intrinsic functionality. However, TCR testing in primary T cells faces an increased degree of variability because of factors such as T‐cell activation status, phenotype or donor origin and is also accompanied by high workloads as well as ethical aspects. Hence, the usage of immortalised T‐cell clones represents an attractive alternative.

The Jurkat leukemic T‐cell line is a widely used model system for the study of TCR function,^21^ and we previously developed a triple parameter TCR signalling reporter cell line (TPR) based on the Jurkat line E6.1.^22^ These reporter cells have been proven to be highly suitable to evaluate co‐stimulatory pathways and the function of chimeric antigen receptors,^23^, ^24^, ^25^ but to date, their potential to evaluate transgenically expressed TCRs in a high‐throughput manner that still reflects physiological T‐cell biology as seen in primary human T cells had not been tested. To facilitate highly sensitive and unbiased TCR functional characterisation, we introduced two additional modifications in the TPR cell line. First, we introduced the CD8αβ co‐receptor as it stabilises the TCR‐peptide major histocompatibility complex (pMHC) interaction and thereby increases the sensitivity of TCR activation.^26^, ^27^, ^28^ Second, since the presence of the endogenous receptor can decrease transgenic TCR functionality^29^, ^30^, ^31^ through competition for CD3 molecules^32^ and/or formation of mixed TCR dimers,^2^, ^33^, ^34^ we performed CRISPR/Cas9‐mediated knockout (KO) of both TCR α‐ and β‐chains. Even with these modifications, however, the suitability of such an immortalised cell line for reliable TCR functional testing was not clear. For instance, Jurkat cells are deficient of PTEN^35^ which potentially alters TCR functionality in comparison to natural TCR function in primary T cells.

Here, we generated CD8αβ^+/−^ endogenous TCR‐deficient TPR cell lines (TPR^KO^‐CD8^−^ and TPR^KO^‐CD8^+^) and comprehensively investigated their suitability for high‐throughput TCR functional testing. In total, we transgenically re‐expressed 59 human TCRs in TPR^KO^ cell lines and performed an in‐depth characterisation of their antigen‐HLA specificity and functional avidity. Most importantly, we also performed these experiments in primary human T cells facilitating direct comparison of TCR function between TPR^KO^ cell lines and primary T cells. We observed that a TCR’s pMHC‐multimer stainability and functional avidity were almost identical in TPR^KO^ cell lines and primary T cells, justifying the usage of our cell line for TCR testing. Furthermore, we document the suitability of TPR^KO^ cell lines for the investigation of TCR biology. Accordingly, we provide further evidence that pMHC‐multimer staining is not directly predictive for TCR functional avidity.^36^, ^37^ Furthermore, by gathering functional TCR data in the presence or absence of CD8αβ, we were able to corroborate previous findings that the CD8αβ co‐receptor increases peptide sensitivity to a highly TCR‐dependent extent^27^, ^28^ and that CD8αβ dependency inversely correlates with TCR functional avidity.^38^, ^39^ Finally, we demonstrate that TPR^KO^ cell lines can be used as the centrepiece of a high‐throughput platform for screening of TCRs for clinical use.

## Results

### **Generation of CD8^+/^** ^−^ **TCR‐replaced Jurkat TCR signal reporter T‐cell lines**

**Figure 1:**
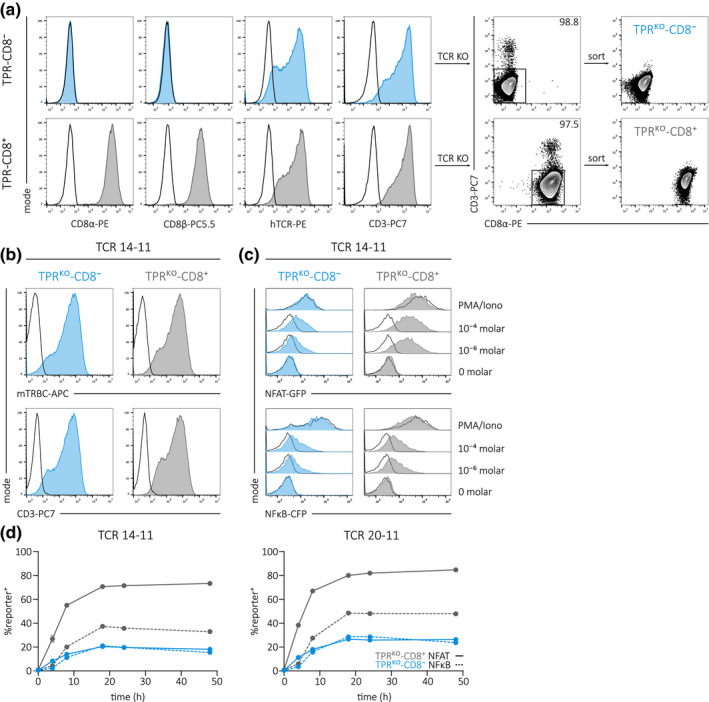
Generation of CD8^+/−^ TCR‐replaced Jurkat TCR signal reporter T‐cell lines. **(a)** Flow cytometry staining of CD8α, CD8β, pan‐human TCR and CD3 of the Jurkat triple parameter cell line^22^ without (TPR‐CD8^−^, blue) or with (TPR‐CD8^+^, grey) transgenic CD8αβ expression. Black line indicates FMO control (left panel). CRISPR/Cas9‐mediated endogenous TCR‐KO (right panel; KO indicated by loss of CD3 expression, numbers indicate KO efficiency). Single cell flow cytometry sorting on CD3‐negative cells and staining after 3 weeks *in vitro* culture (for the gating strategy, see Supplementary figure 1a; for genetic analysis of KO, see Supplementary figure 1b, c). **(b)** Retroviral transduction of TPR^KO^‐CD8^−^ (blue) and TPR^KO^‐CD8^+^ (grey) cells with an A1/pp50_245‐253_‐specific TCR containing murine constant TCR α/β chains. mTRBC staining and re‐expression of CD3 indicate expression of transgenic TCR. Black line represents TCR‐untransduced mock control. **(c)** NFAT and NFκB reporter signal after 24 h of stimulation of TCR 14‐11 expressing TPR^KO^‐CD8^−^ and TPR^KO^‐CD8^+^ cells either with PMA/Iono or A1/pp50 peptide‐pulsed HLA‐A*0101‐positive K562 at indicated concentrations. Black line represents TCR‐untransduced mock control. **(d)** Activation kinetics of NFAT and NFκB in TCR 14‐11 and TCR 20‐11 expressing TPR^KO^‐CD8^−^ and TPR^KO^‐CD8^+^ lines after stimulation with 10^⁻6^ molar A1/pp50 peptide‐pulsed on HLA‐A*0101‐positive K562. For surface expression and stimulation data of TCR 20‐11, see Supplementary figure 2a, b.

We previously reported a highly sensitive TCR signal reporter system based on the T‐cell line Jurkat E6.1^22^ and now aimed to use this cell line for reliable high‐throughput evaluation of TCR function. We additionally introduced CD8 α‐ and β‐chains (Figure 1a, left panel) to increase the sensitivity of our test system since CD8αβ stabilises the TCR‐pMHC interaction and promotes TCR‐mediated signalling.^27^, ^28^, ^40^ As the Jurkat E6.1 cell line expresses an endogenous TCR (as indicated by hTCR and CD3 staining in Figure 1a), we furthermore performed CRISPR/Cas9‐mediated KO of TCR α‐ and β‐chains (Figure 1a, right panel). By that, we eliminated potential interactions between endogenous and transgenic TCRs,^2^, ^29^, ^32^, ^33^, ^34^ which would introduce a fundamental source of bias in our test system. KO efficiency was larger than 97% in both cell lines and single CD3‐negative cells were sorted on a flow cytometer (Figure 1a, right panel; for the gating strategy, see Supplementary figure 1a) .

Subsequently, we validated the full KO of both TCR α‐ and β‐chains via polymerase chain reaction (PCR) and Sanger sequencing of the respective CRISPR/Cas9‐targeted gene regions (Supplementary figure 1b, c). The resulting TPR^KO^‐CD8^−^ and TPR^KO^‐CD8^+^ cell lines were retrovirally transduced with two different A1/pp50_245‐253_‐specific TCRs (containing murine constant regions,^41^ for all TCR sequences, see Supplementary table 1) in order to validate the function of the TCR signal reporter system. The successful introduction of TCRs was indicated by staining of the transgenic TCR with an anti‐murine TCR β constant region antibody (mTRBC) and re‐expression of CD3 (TCR 14–11 in Figure 1b, see TCR 20–11 in Supplementary figure 2a). Transgenic TCR‐expressing TPR^KO^‐CD8^−^ and TPR^KO^‐CD8^+^ cell lines were stimulated for 24 h either with peptide‐pulsed HLA‐A*0101‐positive K562 or phorbol 12‐myristate 13‐acetate (PMA) and ionomycin (Iono). For both cell lines, we observed a peptide‐dose dependent NFAT and NFκB reporter activity as well as strong activation via PMA/Iono (TCR 14–11 in Figure 1c, see TCR 20–11 in Supplementary figure 2b). A comparison between TPR^KO^‐CD8^−^ and TPR^KO^‐CD8^+^ revealed increased reporter signals in the presence of CD8αβ as expected. We further investigated whether the CD8αβ co‐receptor or the introduced transgenic TCR influences the kinetic of NFAT and NFκB reporter expression, as this would compromise results derived from a snapshot analysis at a certain time point. However, reporter kinetics were highly similar between TCRs as well as between TPR^KO^‐CD8^−^ and TPR^KO^‐CD8^+^ cell lines (Figure 1d). We again observed decreased reporter expression in the absence of CD8αβ with maximum reporter signal in both cell lines 18 h after stimulation. In summary, we successfully introduced the CD8αβ co‐receptor and performed CRISPR/Cas9‐mediated endogenous TCR‐KO in a Jurkat E6.1 based TCR signal reporter cell line. Moreover, we validated the function of the reporter system with two transgenically expressed TCRs.

### pMHC‐multimer staining on TPR^KO^ cell lines is reliable and strongly correlates to primary T cells

**Figure 2:**
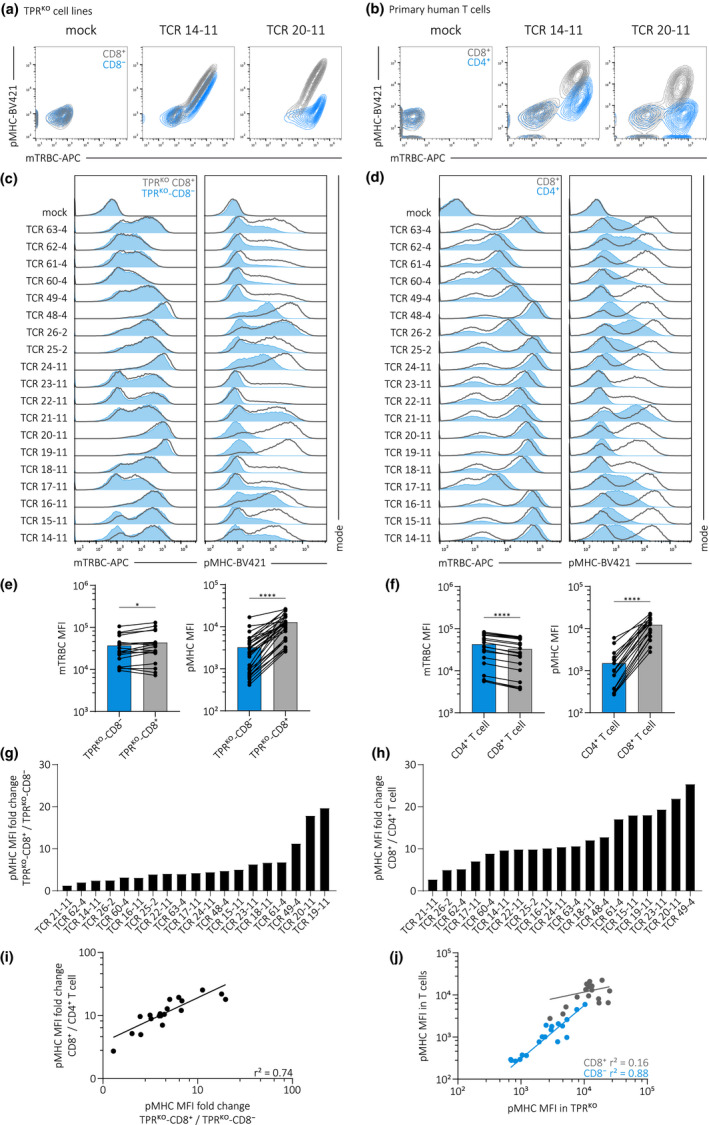
pMHC‐multimer staining on TPR^KO^ cell lines is reliable and strongly correlates to primary T cells. **(a, b)** Flow cytometry mTRBC/pMHC‐multimer co‐staining of two A1/pp50‐specific transgenically expressed TCRs in TPR^KO^ cell lines **(a)** and endogenous TCR‐KO primary T cells **(b)**. TPR^KO^‐CD8^−^ and CD4^+^ primary T cells in blue and TPR^KO^‐CD8^+^ and CD8^+^ primary T cells in grey. **(c, d)** Histograms of mTRBC and pMHC‐multimer staining of 19 A1/pp50‐specific transgenically expressed TCRs in TPR^KO^ cell lines **(c)** and endogenous TCR‐KO primary T cells **(d)**. **(e, f)** Quantification of MFI of mTRBC and pMHC‐multimer staining in TPR^KO^ cell lines **(e)** and endogenous TCR‐KO primary T cells **(f)**. Each dot represents one of 19 individual TCRs. Statistical testing by the two‐tailed paired Student’s *t*‐test, ****P < 0.0001, *P < 0.05. **(g, h)** Quantification of CD8αβ co‐receptor dependency (calculated by fold change of TPR^KO^‐CD8^+^/TPR^KO^‐CD8^−^ or CD8^+^/CD4^+^, respectively) of pMHC‐multimer staining for 19 individual A1/pp50‐specific transgenically expressed TCRs in TPR^KO^ cell lines **(g)** and endogenous TCR‐KO primary T cells **(h)**. **(i)** Correlation of CD8αβ co‐receptor dependency of pMHC‐multimer staining between TPR^KO^ cell lines and primary T cells. Each dot represents one of 19 individual TCRs. Fitting by non‐linear regression. **(j)** Correlation of pMHC‐multimer staining between TPR^KO^ cell lines and primary T cells. Each dot represents one of 19 individual TCRs. Fitting by non‐linear regression.

A first crucial step in TCR functional characterisation is the validation of specific target recognition for which staining with pMHC multimeric complexes^42^ is a particularly efficient method. However, pMHC‐multimer stainings can be a delicate matter. For instance, we observed unsatisfying results with the endogenous TCR‐deficient Jurkat 76 cell line (data not shown). Since reliable pMHC‐multimer staining would facilitate high‐throughput TCR antigen‐HLA specificity screening, we compared TPR^KO^ cell lines and primary human T cells in this respect. For this, we introduced TCR 14–11 and TCR 20–11 in primary human T cells and additionally performed KO of the endogenous TCR α‐ and β‐chains. We observed highly similar pMHC‐multimer staining in our TPR^KO^ cell lines (Figure 2a) in comparison with primary T cells (Figure 2b). Both TCRs, in TPR^KO^ cell lines as well as in primary T cells, showed increased staining intensity in the presence of CD8αβ as expected.^27^ Interestingly, the two transgenically expressed TCRs in TPR^KO^‐CD8^−^ or CD4^+^ primary T cells showed largely different pMHC‐multimer staining intensity, indicating differential dependency on the CD8αβ co‐receptor for pMHC‐multimer binding. To further validate the applicability of our cell lines for pMHC‐multimer staining and to investigate the TCR‐intrinsic ability to bind pMHC‐multimer in the presence and absence of CD8αβ, we introduced 19 different A1/pp50‐specific TCRs in TPR^KO^ cell lines and endogenous TCR‐KO primary T cells. For all 19 TCRs, we observed high transduction efficiencies (indicated by mTRBC staining) and highly similar pMHC‐multimer stainings between TPR^KO^ cell lines (Figure 2c) and CD4^+^/CD8^+^ primary T cells (Figure 2d). pMHC‐multimer staining of individual TCRs was largely variable, particularly in absence of CD8αβ as observed before.^38^, ^39^ Quantification of pMHC‐multimer staining mean fluorescence intensity (MFI) revealed that CD8αβ significantly increases pMHC‐multimer staining in TPR^KO^ cell lines (Figure 2e) and primary T cells (Figure 2f). TCR surface expression was marginally increased in TPR^KO^‐CD8^+^ but decreased in primary human CD8^+^ T cells, presumably reflecting slightly different transduction efficiencies and not being generally related to CD8. Weak correlation of mTRBC MFI with pMHC‐multimer MFI in TPR^KO^ cell lines (Supplementary figure 3a) and primary T cells (Supplementary figure 3b) indicates that pMHC‐multimer stainability is not a mere function of TCR surface expression level but a TCR‐intrinsic feature. Furthermore, we observed a large spectrum of different dependencies on the CD8αβ co‐receptor as quantified by pMHC‐multimer MFI fold changes (Figure 2g, h), whereas we did not observe such different dependencies on CD8αβ for TCR surface expression (Supplementary figure 3c, d). Most importantly, we observed strong correlations between TPR^KO^ cell lines and primary T cells regarding CD8αβ dependency (Figure 2i) and pMHC‐multimer staining intensity (Figure 2j). In case of the latter, the correlation was particularly strong in the absence of CD8αβ, indicating that inter‐TCR differences in pMHC‐multimer staining are to some extent masked by the CD8αβ contribution to the TCR‐pMHC interaction. In summary, we observed highly reliable pMHC‐multimer staining with our TPR^KO^ cell lines that strongly correlates to primary T cells. Using TPR^KO^‐CD8^−^ and TPR^KO^‐CD8^+^ cells for pMHC‐multimer stainings of 19 individual transgenically expressed TCRs further validated a significant contribution of CD8αβ to the stability of the TCR‐pMHC complex and revealed a large spectrum of TCR‐intrinsic pMHC‐multimer stainability, particularly in the absence of CD8αβ.

### TPR^KO^ cell lines facilitate high‐resolution assessment of TCR functionality

**Figure 3:**
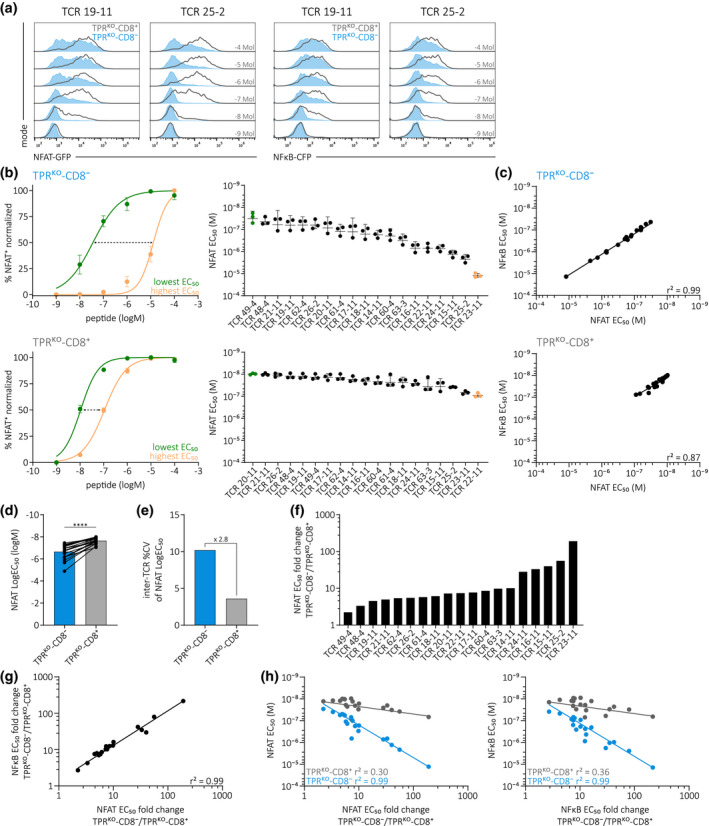
TPR^KO^ cell lines facilitate high‐resolution assessment of TCR functionality. **(a)** NFAT and NFκB reporter signal of two A1/pp50‐specific transgenically expressed TCRs in TPR^KO^ cell lines 18h after stimulation with indicated concentrations of peptide, pulsed on HLA‐A*0101‐positive K562. TPR^KO^‐CD8^−^ in blue and TPR^KO^‐CD8^+^ in grey. **(b)** NFAT reporter EC_50_ curves of most (lowest EC_50_, green) and least (highest EC_50_, orange) antigen‐sensitive TCRs (left) and quantification of EC_50_ of 19 A1/pp50‐specific transgenically expressed TCRs in TPR^KO^‐CD8^−^ (upper panel) and TPR^KO^‐CD8^+^ (lower panel) cell lines. Stimulation assay as in **(a)**. Dashed arrows indicate range between lowest and highest EC_50_. Depicted are replicates and mean ± s.d. **(c)** Correlation of NFAT (shown in b) to NFκB reporter EC_50_ (shown in Supplementary figure 4a) in TPR^KO^‐CD8^−^ (top) and TPR^KO^‐CD8^+^ (bottom) cell lines. Fitting by non‐linear regression. **(d)** Comparison of NFAT reporter LogEC_50_ of 19 A1/pp50‐specific transgenically expressed TCRs in TPR^KO^ cell lines with and without CD8αβ co‐receptor. Each dot represents one of 19 individual TCRs. Statistical testing by the two‐tailed paired Student’s *t*‐test, ****P < 0.0001. **(e)** Quantification of NFAT LogEC_50_ variability between 19 A1/pp50 TCRs. Fold change between TPR^KO^ cell lines is indicated. **(f)** Quantification of CD8αβ co‐receptor dependency (calculated by fold change of TPR^KO^‐CD8^−^/TPR^KO^‐CD8^+^; high value represents high dependency) of NFAT reporter EC_50_ for 19 individual A1/pp50‐specific transgenically expressed TCRs in TPR^KO^ cell lines. Each bar represents the mean of three replicates. **(g)** Correlation of NFAT reporter EC_50_ CD8αβ co‐receptor dependency and NFκB reporter EC_50_ CD8αβ co‐receptor dependency. Fitting by non‐linear regression. **(h)** Correlation of NFAT reporter EC_50_ CD8αβ co‐receptor dependency to NFAT reporter EC_50_ (left) and NFκB reporter EC_50_ CD8αβ co‐receptor dependency to NFκB reporter EC_50_ (right). Each dot represents one of 19 individual TCRs. Fitting by non‐linear regression.

TPR^KO^ cell lines can thus be used to systematically screen a large number of TCRs for antigen‐HLA specificity via pMHC‐multimer staining. As a next step, we investigated the suitability of our cell lines for the assessment of TCR functional avidity. For this, we performed antigen‐specific stimulation with peptide‐pulsed HLA‐A*0101 K562 and measured NFAT and NFκB reporter activity after 18 h. We observed a peptide‐dose dependent reporter response in both TPR^KO^ cell lines for two individual A1/pp50‐specific transgenically expressed TCRs (Figure 3a). As observed before (Figure 1c), the reporter signal was increased in the presence of CD8αβ, but yet again, to a different extent between TCRs. To investigate TCR functionality in our TPR^KO^ cell lines in more detail, we performed peptide titrations with 19 A1/pp50‐specific TCRs. TPR^KO^ cell lines facilitated the measurement of dose‐response curves with very minor technical and/or biological variability (Figure 3b, left), indicating that TCR functional avidity can be assessed with high reliability and resolution. Based on the dose‐response curves, we calculated half‐maximal effective concentrations (EC_50_) of NFAT reporter activity (Figure 3b, right). Similarly to pMHC‐stainability (Figure 2), we observed a large spectrum of functional avidities in the absence of CD8αβ (Figure 3b, upper panel). In the presence of CD8αβ (Figure 3b, lower panel), peptide sensitivity was significantly increased for all 19 TCRs (Figure 3d), but functional differences between TCRs were distinctly smaller as indicated by a decreased coefficient of variation between TCRs (Figure 3e). NFκB reporter responses (Supplementary figure 4a–c) were highly similar to NFAT reporter signals as indicated by correlations between NFAT EC_50_ and NFκB EC_50_ for both TPR^KO^ cell lines (Figure 3c) The presence of CD8αβ also significantly increased the maximal responsiveness to antigen (E_max_) (Supplementary figure 4d). Again, we could observe a large spectrum of different CD8αβ co‐receptor dependencies regarding NFAT EC_50_ (Figure 3f) and NFκB EC_50_ (Supplementary figure 4e), with both measurements strongly correlating to each other (Figure 3g). Correlations between TPR^KO^‐CD8^−^ and TPR^KO^‐CD8^+^ for NFAT EC_50_ (Supplementary figure 4f) and NFκB EC_50_ (Supplementary figure 4g) were not strong, mainly because of the small functional differences between TCRs in the presence of CD8αβ but also indicating TCR‐intrinsic CD8αβ co‐receptor dependency. Interestingly, the correlation of CD8αβ dependency to functional avidity revealed an inverse correlation that was particularly strong in TPR^KO^‐CD8^−^ (Figure 3h). In summary, TPR^KO^ cell lines facilitate TCR functional characterisation with high resolution and low technical and/or biological variability. TCR‐intrinsic differences in functional avidity are particularly visible in the absence of CD8αβ. Furthermore, co‐receptor dependency inversely correlates to functionality. Hence, low avidity TCRs disproportionally benefit from CD8αβ, whereas high avidity TCRs show only little additional gain in peptide sensitivity.

### TCR functional avidity determined in TPR^KO^ cell lines strongly correlates to primary T cells

**Figure 4:**
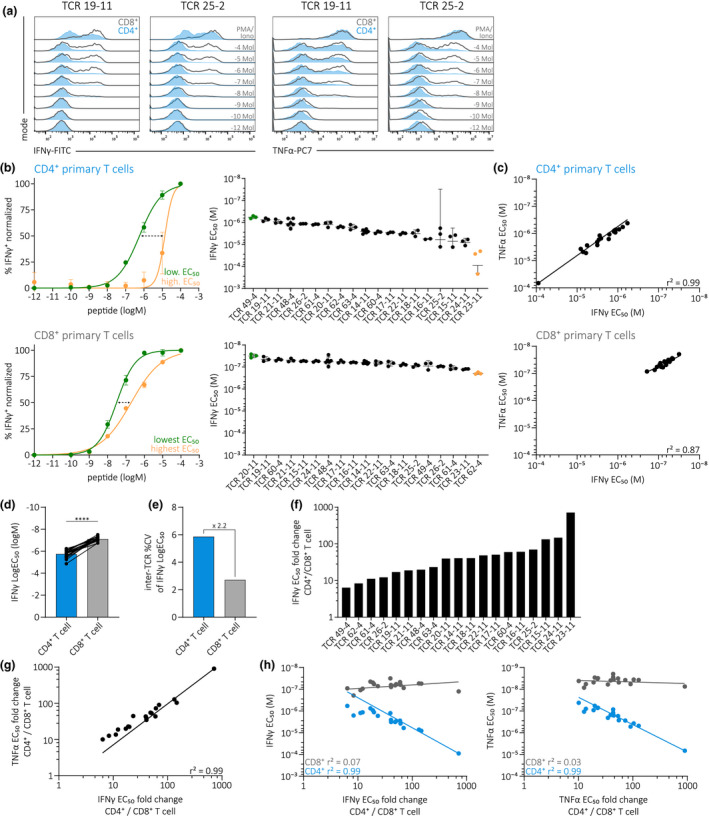
Determination of TCR functionality in CD4^+^/CD8^+^ primary T cells. **(a)** Intracellular staining of IFNγ (left) and TNFα (right) expression of two A1/pp50‐specific transgenically expressed TCRs in endogenous TCR‐KO primary T cells 4h after stimulation with indicated concentrations of peptide, pulsed on HLA‐A*0101‐positive K562. CD4^+^ primary T cells in blue and CD8^+^ primary T cells in grey. **(b)** IFNγ EC_50_ curves of most (lowest EC_50_, green) and least (highest EC_50_, orange) antigen‐sensitive TCRs (left) and quantification of EC_50_ of 19 A1/pp50‐specific transgenically expressed TCRs in CD4^+^ (upper panel) and CD8^+^ (lower panel) endogenous TCR‐KO primary T cells. Stimulation assay as in **(a)**. Dashed arrows indicate range between lowest and highest EC_50_. Depicted are replicates and mean ± s.d. **(c)** Correlation of IFNγ (shown in b) to TNFα EC_50_ (shown in Supplementary figure 5a) in CD4^+^ (upper panel) and CD8^+^ (lower panel) endogenous TCR‐KO primary T cells. Fitting by non‐linear regression. **(d)** Comparison of IFNγ LogEC_50_ of 19 A1/pp50‐specific transgenically expressed TCRs in CD4^+^ and CD8^+^ endogenous TCR‐KO primary T cells. Each dot represents one of 19 individual TCRs. Statistical testing by the two‐tailed paired Student’s *t*‐test, ****P < 0.0001. **(e)** Quantification of IFNγ LogEC_50_ variability between 19 A1/pp50 TCRs. Fold change between CD4^+^ and CD8^+^ T cells is indicated. **(f)** Quantification of CD8αβ co‐receptor dependency (calculated by fold change of CD4^+^/CD8^+^; high value represents high dependency) of IFNγ reporter EC_50_ for 19 individual A1/pp50‐specific transgenically expressed TCRs in endogenous TCR‐KO primary T cells. Each bar represents the mean of three replicates. **(g)** Correlation to IFNγ reporter EC_50_ CD8αβ co‐receptor dependency and TNFα reporter EC_50_ CD8αβ co‐receptor dependency. Fitting by non‐linear regression. **(h)** Correlation of IFNγ EC_50_ CD8αβ co‐receptor dependency to IFNγ EC_50_ (left) and TNFα EC_50_ CD8αβ co‐receptor dependency to TNFα EC_50_ (right). Each dot represents one of 19 individual TCRs. Fitting by non‐linear regression.

We have demonstrated that TPR^KO^ cell lines can be used for large scale assessment of TCR functional avidity. However, we were concerned whether these TCR functionality data accurately reflect data obtained with primary human T cells. While the Jurkat cell line represents a generally accepted model system for investigation of T‐cell activation and TCR signalling, there might also be TCR function affecting differences between this immortalised cell line and primary T cells, such as a reported PTEN deficiency^35^. Therefore, our goal was to systematically compare TCR functionality in TPR^KO^ cell lines to primary human T cells. For this, we introduced the same 19 A1/pp50‐specific TCRs (shown in Figure 3) into endogenous TCR‐KO primary CD4^+^/CD8^+^ T cells and performed intracellular cytokine staining of interferon gamma (IFNγ) and tumor necrosis factor alpha (TNFα) upon antigen‐specific stimulation with peptide‐pulsed HLA‐A*0101 K562. In general, investigation of transgenically expressed TCRs in primary T cells revealed highly similar relations between TCR functional avidity and CD8αβ co‐receptor contribution as observed in TPR^KO^ cell lines: peptide sensitivity and E_max_ were increased in CD8^+^ compared to CD4^+^ primary T cells (Figure 4a–d and Supplementary figure 5 a–d); TCR‐intrinsic differences in functional avidity were particularly visible in CD4^+^ T cells (Figure 4b, e and Supplementary figure 5a, c); IFNγ and TNFα responses strongly correlated with each other (Figure 4c); IFNγ (Figure 4f) and TNFα (Supplementary figure 5e) EC_50_ CD8αβ co‐receptor dependency were largely variable between TCRs and strongly correlated between IFNγ and TNFα (Figure 4g); correlations between CD4^+^ and CD8^+^ primary T cells for IFNγ EC_50_ (Supplementary figure 5f) and TNFα EC_50_ (Supplementary figure 5g) were not strong as observed in TPR^KO^ cells; and IFNγ and TNFα EC_50_ inversely correlated to CD8αβ co‐receptor dependency, which was particularly strong in CD4^+^ T cells (Figure 4h).

**Figure 5:**
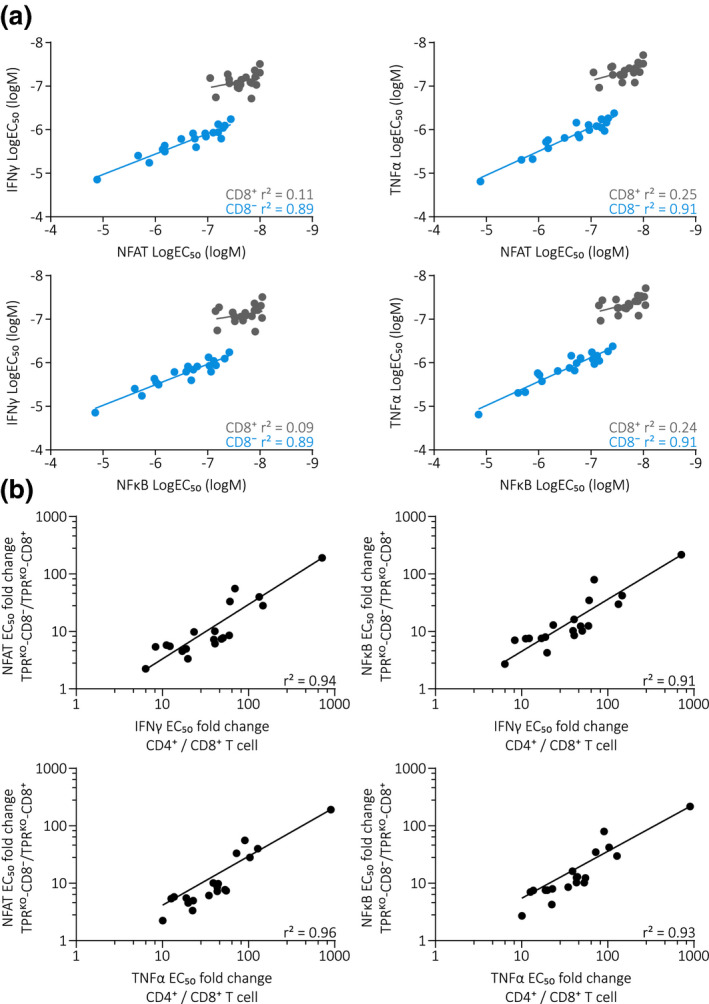
TCR functional avidity determined in TPR^KO^ cell lines strongly correlates to primary T cells. **(a)** Correlation of NFAT (upper panel) and NFκB (lower panel) reporter LogEC_50_ measured in TPR^KO^ cell lines to IFNγ (left) and TNFα (right) LogEC_50_ measured in endogenous TCR‐KO primary T cells. Each dot represents one of 19 individual A1/pp50‐specific transgenically expressed TCRs. Fitting by linear regression. **(b)** Correlation of IFNγ (upper panel) and TNFα (lower panel) EC_50_ CD8αβ co‐receptor dependency measured in endogenous TCR‐KO primary T cells to NFAT (left) and NFκB (right) reporter EC_50_ CD8αβ co‐receptor dependency measured in TPR^KO^ cell lines. Each dot represents one of 19 individual A1/pp50‐specific transgenically expressed TCRs. Fitting by non‐linear regression.

Direct comparison of NFAT and NFκB EC_50_ values measured in TPR^KO^ cell lines with IFNγ and TNFα EC_50_ values measured in CD4^+^/CD8^+^ primary T cells revealed a surprisingly strong correlation, particularly in the absence of CD8αβ co‐receptor (Figure 5a), indicating that inter‐TCR differences are masked by CD8αβ contribution. CD8αβ co‐receptor dependency of functional avidity was also strongly correlating between TPR^KO^ cell lines and primary T cells (Figure 5b). We further related functional avidity data to pMHC‐multimer staining data and did not observe a correlation in both TPR^KO^ cell lines (Supplementary figure 6a) and primary T cells (Supplementary figure 6b), neither for CD8^+^ nor for CD8^−^ cells. Accordingly, CD8αβ co‐receptor dependency of functional avidity and CD8αβ co‐receptor dependency of pMHC‐multimer staining also did not correlate in TPR^KO^ cell lines (Supplementary figure 6c) and primary T cells (Supplementary figure 6d). These findings generated with a plethora of different TCRs systematically side‐by‐side are in line with previous reports that document no or at most a very limited correlation between pMHC‐multimer stainability and TCR functionality.^36^, ^37^ Interestingly, these data further indicate that CD8αβ contributes to pMHC‐multimer staining and functional avidity via different mechanisms.

Most importantly, we show that TCR functional avidity in TPR^KO^ cell lines strongly parallels TCR functional avidity in primary T cells. Hence, TPR^KO^ cell lines can be used as a surrogate of primary T cells, which facilitates a high‐throughput, standardised and reliable characterisation of TCR functional avidity. Furthermore, our data on the relations of CD8αβ co‐receptor to pMHC‐multimer staining and functional avidity illustrate the suitability of our TPR^KO^ cell lines for investigation of TCR biology in general.

### TPR^KO^ cell lines as the centrepiece of a high‐throughput TCR screening platform

**Figure 6:**
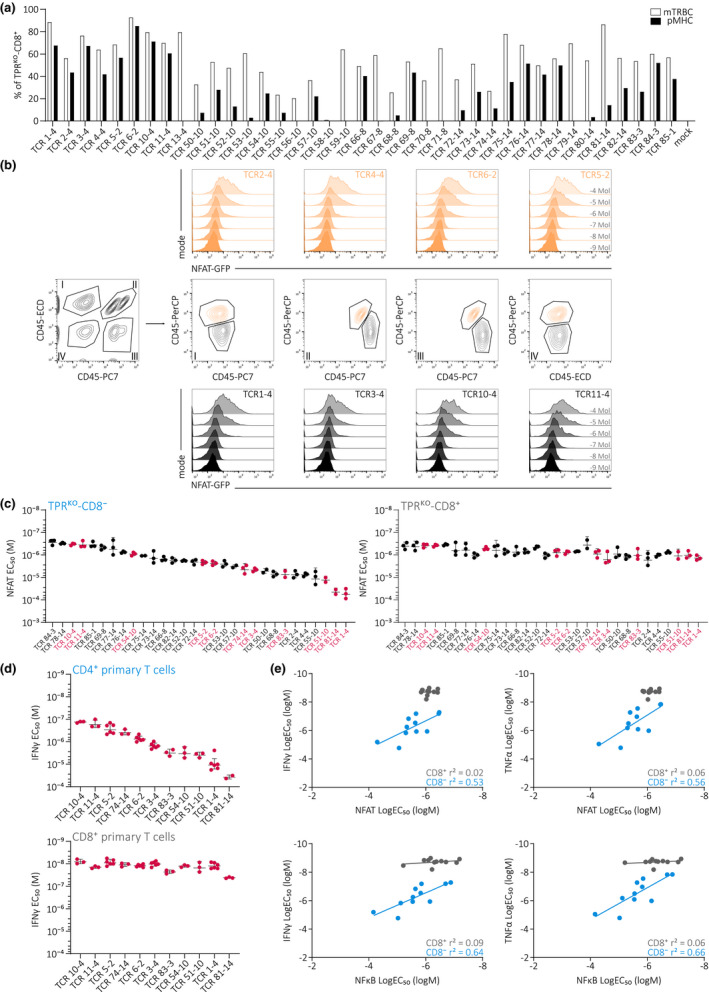
TPR^KO^ cell lines as the centrepiece of a high‐throughput TCR screening platform. **(a)** Quantification of transgenic TCR surface expression (mTRBC, white bars) and pMHC‐multimer staining (pMHC, black bars) of 38 transgenically expressed TCRs in TPR^KO^‐CD8^+^ cells. TCRs were initially isolated by flow cytometry sorting of A2/pp65_495‐593_ pMHC‐multimer^+^ CD8^+^ T cells. **(b)** High‐throughput TCR functional testing in TPR^KO^ cell lines using a triple CD45 antibody‐based colour code that enables measurement of eight samples at once. Each sample (i.e. a certain TCR transgenically expressed in a TPR^KO^ cell line) is stained with a unique code of CD45‐ECD, CD45‐PC7 and CD45‐PerCP antibodies. Shown is NFAT reporter signal of 8 different A2/pp65‐specific TCRs in TPR^KO^. Roman numerals indicate colour code gating. **(c)** Quantification of NFAT reporter EC_50_ of 30 A2/pp65‐specific TCRs in TPR^KO^‐CD8^−^ (left) and TPR^KO^‐CD8^+^ (right). Eleven TCRs marked in red were selected for further functional testing in primary T cells. TCRs are ordered from left to right according to NFAT EC_50_ in TPR^KO^‐CD8^−^. Depicted are replicates and mean ± s.d. Quantification of NFκB EC_50_ is shown in Supplementary figure 8a. **(d)** Quantification of IFNγ EC_50_ of eleven selected A2/pp65‐specific TCRs in CD4^+^ (top) and CD8^+^ (bottom) endogenous TCR‐KO primary T cells. TCRs are ordered from left to right according to IFNγ EC_50_ in CD4^+^ primary T cells. Depicted are replicates and mean ± s.d. Quantification of TNFα EC_50_is shown Supplementary figure 8b. **(e)** Correlation of NFAT (upper panel) and NFκB (lower panel) reporter LogEC_50_ measured in TPR^KO^ cell lines to IFNγ (left) and TNFα (right) LogEC_50_ measured in endogenous TCR‐KO primary T cells. Each dot represents one of eleven individual A2/pp65‐specific TCRs. Fitting by linear regression.

In order to validate the suitability of our TPR^KO^ cell lines for high‐throughput and reliable determination of TCR antigen‐HLA specificity and functionality, we tested our system with 38 TCRs that were initially isolated by flow cytometry sorting of A2/pp65_495‐593_ pMHC‐multimer^+^ CD8^+^ T cells. First, we performed retroviral transduction of all 38 TCRs into TPR^KO^‐CD8^+^ cells to determine TCR surface expression and antigen‐HLA specificity via pMHC‐multimer staining. 30 TCRs could be re‐stained with pMHC‐multimer, whereas seven TCRs did not stain with pMHC‐multimer (TCRs 13–4, 56–10, 59–10, 67–8, 70–8, 71–8, and 79–14) and one TCR was not expressed at all on the cell surface (TCR 58–10) (Figure 6a). For TCR 13–4, we confirmed the lack of antigen‐HLA specificity in primary T cells (Supplementary figure 7). Having identified 30 A2/pp65‐specific TCRs, we subsequently determined their functional avidity. In order to streamline the measurement of 30 TCRs upon stimulation with six different peptide concentrations in triplicates (equals 540 samples), we performed multiplexing via a CD45 antibody barcoding approach. Using combinations of three differently fluorochrome‐labelled CD45 antibodies, eight individual samples receive a unique colour barcode and can thereby be pooled within one sample (Figure 6b). The sample number was thereby reduced to 72. Usage of additional CD45 antibodies with different fluorochrome labels could have easily further decreased this number. Quantification of NFAT (Figure 6c) and NFκB (Supplementary figure 8a) EC_50_ values revealed a large spectrum of different TCR functional avidities, particularly in absence of CD8αβ as observed before with A1/pp50‐specific TCRs (Figure 3b and Supplementary figure 4a). Based on NFAT EC_50_ values measured in TPR^KO^‐CD8^−^ cells, we selected eleven TCRs, covering the whole avidity spectrum (Figure 6c, marked in red colour), for TCR re‐expression and functional characterisation in primary human T cells. Again, we observed a large spectrum of IFNγ (Figure 6d) and TNFα (Supplementary figure 8b) EC_50_ values in CD4^+^ T cells, whereas this diversity was decreased in CD8^+^ T cells. Between TPR^KO^ cell lines and primary T cells, the functionality of these eleven A2/pp65‐specific TCRs correlated well in absence, but not in presence of CD8αβ (Figure 6e). Finally, we also compared pMHC‐multimer staining of these TCRs in TPR^KO^ cell lines (Supplementary figure 9a) and endogenous TCR‐KO primary T cells (Supplementary figure 9b). CD8αβ co‐receptor presence increased pMHC‐multimer staining both in TPR^KO^ cell lines (Supplementary figure 9c) as well as in endogenous TCR‐KO primary T cells (Supplementary figure 9d). CD8αβ dependency (Supplementary figure 9e) and pMHC‐multimer stainability (Supplementary figure 9f) of TCRs strongly correlated between TPR^KO^ cell lines and primary T cells, the latter especially in absence of CD8αβ as observed with A1/pp50‐specific TCRs (Figure 2j). In summary, we here provide proof‐of‐concept for the suitability of our TPR^KO^ cell lines for high‐throughput and reliable screening of TCR antigen‐HLA specificity and functional avidity.

## Discussion

The functional characterisation of TCRs is most widely performed after transgenic re‐expression in primary T cells. Variability in primary T cells because of phenotype, activation status, or donor origin can affect TCR function and bias results. Hence, a cell line that provides TCR function close to primary T cells would enable more standardised testing as well as simplify the whole process because of cell lines’ easy handling and almost unlimited proliferative capacity. The urgent need for such a cell line is highlighted by various publications that proposed different cellular platforms for TCR testing.^43^, ^44^, ^45^, ^46^, ^47^

Here, we propose an advanced Jurkat E6.1‐based TCR signal reporter system that is unbiased by endogenous TCR expression. Our study, which analysed 59 different human TCRs, is – to our knowledge – the first to comprehensively compare TCR function in a cell line with primary human T cells. As TCR function was closely parallel to primary T cells, our TPR^KO^ cell lines proved highly suitable for functional characterisation of individual TCRs and also for the investigation of TCR biology in general. By relating functional avidity to pMHC‐multimer staining data, both in TPR^KO^ cell lines and primary T cells (Supplementary figure 6), we validated that pMHC‐multimer staining intensity is not predictive for functionality,^36^, ^48^ highlighting the importance of functional testing for the identification of suitable TCRs for clinical use. Further, we confirmed previous findings that the CD8αβ co‐receptor increases a TCR’s peptide sensitivity to a highly differential TCR‐dependent extent^27^, ^28^ and CD8αβ co‐receptor dependency inversely correlates with functional avidity.^38^, ^39^, ^49^ The latter implicates that measured TCR functional avidity in absence of CD8αβ might more directly reflect the structural avidity of a TCR to its cognate pMHC. We further observed a disparity between CD8αβ dependency of pMHC‐multimer staining and of TCR functional avidity, indicating the presence of two different mechanisms of CD8αβ contribution to pMHC‐multimer binding and antigen‐specific TCR activation. Our TPR^KO^ cell lines could be used as a tool to investigate this more closely.

Whereas TCR sequencing^10^, ^11^, ^12^, ^13^ and antigen‐HLA specificity prediction algorithms^14^, ^15^ are in constant progress, validation of TCR specificity and function remains a bottleneck. On this aspect, recently reported protocols for high‐throughput direct cloning of TCRs for transgenic re‐expression represent major progress for large scale TCR re‐expression.^12^, ^13^, ^50^ We here document highly reliable pMHC‐multimer staining on our TPR^KO^ cell lines demonstrating their suitability for large scale antigen‐HLA specificity screening approaches. For instance, this enables re‐expression of large combinatorial libraries of TCR α‐ and β‐chains in our TPR^KO^ cell lines for high‐throughput screening of antigen‐HLA specificities of interest. Furthermore, we have demonstrated that TPR^KO^ cell lines facilitate a high‐throughput functionality screening of TCRs with high sensitivity and reliability. Hence, TPR^KO^ cell lines enable the generation of large datasets connecting TCR sequence, antigen‐HLA specificity, and function to an unprecedented extent. This would be a substantial contribution to the development of improved algorithms for antigen‐HLA specificity and probably also functionality prediction from raw TCR sequence data.^14^, ^15^, ^51^ Fast sequencing of TCR repertoires in combination with such reliable prediction algorithms has the potential to revolutionise patient‐individualised adoptive T‐cell therapy.

Determination of TCR functionality in the presence and absence of CD8αβ enables identification of largely CD8αβ co‐receptor‐independent TCRs, which could be of particular interest for clinical application. On the one hand, CD8αβ‐independent TCRs would maintain their functionality in T‐cell products despite largely variable CD8αβ expression.^39^ On the other hand, it was shown that CD4^+^ T cells expressing an MHC class I‐restricted TCR provide important additional TCR functions, such as increased IL‐2 help, and thereby contribute to an increased anti‐tumor response.^52^, ^53^, ^54^ Hence, CD8αβ co‐receptor‐independent TCRs would represent ideal candidates for such an approach.

In summary, we here propose a Jurkat‐based TCR signal reporter cell line for testing of TCR specificity and functionality unbiased by endogenous TCR expression. TCR functional avidity of 30 individual TCRs in our TPR^KO^ cell lines was strongly correlating to primary human T cells, highlighting the suitability of our cell line for highly reliable investigation of TCR function and biology. Hence, this platform represents a valuable tool for the characterisation and selection of TCR candidates for clinical use and also facilitates the generation of large TCR functionality datasets for the development of prediction algorithms.

## Methods

### Cell culture

TPR^KO^ cell lines and primary T cells were cultured in RPMI 1640 (Gibco, Thermo Fisher Scientific; Waltham, Massachusetts) supplemented with 10 % FCS, 0.025 % L‐glutamine, 0.1 % HEPES, 0.001 % gentamycin and 0.002 % streptomycin ('RPMI' hereafter). Primary T‐cell culture was additionally supplemented with 180 IU mL^−1^ IL‐2.

Written informed consent was obtained from peripheral blood mononuclear cell (PBMC) donors, and usage of the blood samples was approved according to national law by the local Institutional Review Board (Ethikkommission der Medizinischen Fakultät der Technischen Universität München). The study conforms to the standards of the Declaration of Helsinki.

### TCR identification

PBMCs of CMV‐seropositive, healthy donors were stained with respective pMHC‐multimer that was individually conjugated with two different fluorophores to achieve reliable double pMHC‐multimer staining. Single cells positive for CD8, CD62L, CD45RO, and both pMHC‐multimer conjugates were sorted in a 384‐well plate and stimulated with 10 µg mL^−1^ plate‐bound anti‐CD3 and anti‐CD28 each. RPMI medium was supplemented with 200 IU mL^−1^ IL‐2 and 5 ng mL^−1^ IL‐15. Single cell‐derived clones were harvested between days 7 and 14 after sorting. TCRs were amplified via TCR‐SCAN RACE PCR^55^ and subsequently sequenced on the Illumina MiSeq platform. TCR nomenclature represents a consecutive numbering with no meaning for the here presented data.

### TCR DNA template design

DNA templates were designed *in silico* and synthesised by GeneArt (Life Technologies, Thermo Fisher Scientific) or Twist Bioscience (San Francisco, California). DNA constructs for retroviral transduction had the following structure: Human Kozac sequence^56^ followed by TCR β (including a murine TCR β constant region (TRBC) with additional cysteine bridge^41^, ^57^, ^58^), followed by P2A and followed by TCR α (including a murine TCR α constant region (TRAC) with additional cysteine bridge^41^, ^57^, ^58^), cloned into pMP71 vectors (kindly provided by Wolfgang Uckert, Berlin).

### Cas9 RNPs

crRNA sequences for gRNAs were 5′‐GGAGAATGACGAGTGGACCC‐3′ for TRBC^59^ (targeting both TRBC1 and TRBC2) and 5′‐AGAGTCTCTCAGCTGGTACA‐3′ for TRAC.^59^ 80 µM tracrRNA (IDT DNA; Coralville, Iowa) and 80 µM crRNA (IDT DNA) were incubated at 95°C for 5 min, then cooled to RT on the benchtop. 24 µM high‐fidelity Cas9 (IDT DNA) was added slowly to gRNA solution to yield RNPs with 12 µM Cas9 and 20 µM gRNA, as well as 20 µM electroporation enhancer (IDT DNA). RNPs were incubated for 15 min at RT.

### CRISPR/Cas9‐mediated KO

Bulk PBMCs were activated for two days with CD3/CD28 Expamer (Juno therapeutics a Bristol‐Myers Squibb Company; Seattle, Washington), 300 IU mL^−1^ IL‐2, 5 ng mL^−1^ IL‐7 and 5 ng mL^−1^ IL‐15 per ml RPMI for 1 × 10^6^ T cells. Expamer stimulus was removed by incubation with 1 mM D‐biotin. 1 × 10^5^ mL^−1^ TPR cells were seeded in a 24‐well plate two days before electroporation. Cells were electroporated (pulse code EH‐100 for primary T cells and CL‐120 for TPR cells) with Cas9 ribonucleoprotein in Nucleofector Solution (20 µL per 1 × 10^6^ cells; Lonza; Basel, Switzerland) with a 4D Nucleofector X unit (Lonza). After electroporation, cells were cultured in RPMI with 180 IU mL^−1^ IL‐2 (primary T cells) or RPMI without supplements (TPR cells) until a first FACS analysis on day five after editing.

### Retroviral transduction

Retroviral transduction of TPR^KO^ cell lines and primary human T cells was performed using the RD114 virus packaging cell line. For the production of retroviral particles, RD114 cells were transfected with pMP71 expression vector (containing the TCR construct) by calcium phosphate precipitation. Virus supernatant was harvested after 72 h and subsequently coated on retronectin‐treated (TaKaRa; Kusatsu, Japan) well plates. Bulk PBMCs were activated for two days with CD3/CD28 Expamer (Juno therapeutics a Bristol‐Myers Squibb Company), 300 IU mL^−1^ IL‐2, 5 ng mL^−1^ IL‐7 and 5 ng mL^−1^ IL‐15 per mL of RPMI for 1 × 10^6^ T cells. Expamer stimulus was removed by incubation with 1 mM D‐biotin. 1 × 10^5^ mL^−1^ TPR cells were seeded in a 24‐well plate two days before transduction. Activated T cells or TPR cells were transduced via spinoculation on virus‐coated plates. TCR transduction occurred 15 min after CRISPR/Cas9‐mediated TCR‐KO editing of T cells.

### pMHC‐multimer and antibody staining

pMHC‐monomers were generated as previously described.^60^ All biotinylated pMHC‐monomers were multimerised by incubation of 4 µg biotinylated pMHC monomer with 1 µg streptavidin‐BV421 (BioLegend; San Diego, California) or streptavidin‐PE (BioLegend) in a total volume of 100 µL FACS buffer per 1 x 10^7^ cells. The following antibodies were used: anti‐human TCR α/β PE (BioLegend), CD3 PC7 (BD Biosciences; San Jose, California), CD8α PE (Invitrogen, Thermo Fisher Scientific), CD8β PC5.5 (Beckman Coulter; Brea, California), CD45 PerCP (Thermo Fisher Scientific), CD45 ECD (Beckman Coulter), CD45 PC7 (eBioscience, Thermo Fisher Scientific) and anti‐mTRBC APC (Biolegend). Live/dead discrimination was performed with propidium iodide (Invitrogen).

### Antigen‐specific activation and intracellular cytokine staining

One day before co‐culture with T cells, K562 cells (retrovirally transduced to express the MHC class I molecule of interest) were irradiated (80 Gy) and loaded with peptide (10^−12^ M, 10^−10^ M, 10^−9^ M, 10^−8^ M, 10^−7^ M, 10^−6^ M, 10^−5^ M, 10^−4^ M) overnight at 37°C. T cells were co‐cultured with peptide‐loaded K562 cells and Golgi plug (BD Biosciences) in a 1:1 ratio for 4 h at 37°C. PMA (25 ng mL^−1^) and Iono (1 µg mL^−1^) were used for positive control. pMHC‐multimer and surface marker antibody staining for CD8α (PE, Invitrogen) and anti‐mTRBC (APC, BioLegend) was followed by permeabilisation using Cytofix/Cytoperm (BD Biosciences), and staining of IFNγ (FITC, BD Biosciences) and TNFα (PC7, eBioscience). Live/dead discrimination was performed with ethidium‐monoazide‐bromide (Invitrogen).

### Antigen‐specific activation and TCR signalling

TCRs were introduced into the TPR^KO^ cell lines via retroviral transduction. Antigen‐specific stimulation was performed using irradiated (80 Gy) and peptide‐pulsed (10^−9^ M, 10^−8^ M, 10^−7^ M, 10^−6^ M, 10^−5^ M, 10^−4^ M) K562 cells (retrovirally transduced to express the MHC class I molecule of interest). Effector and target cells were co‐cultured in a 1:5 ratio for 18 h. Subsequently, NFAT‐GFP and NFκB‐CFP reporter expression was analysed on a flow cytometer.

### Sanger sequencing for KO validation

Genomic DNA was extracted (Wizard SV Genomic DNA Purification System, Promega; Madison, Wisconsin) from flow‐sorted CD3‐negative TPR cells. PCRs were performed to amplify the intended CRISPR/Cas9‐mediated cutting sites within the first exon of TRAC as well as the first exon of TRBC1/2. Purified PCR products were Sanger sequenced (Eurofins Genomics, Ebersberg, Germany).

### Flow cytometry

Acquisition of FACS samples was done on a Cytoflex (S) flow cytometer (Beckman Coulter). Flow sorting was conducted on a FACSAria III (BD Bioscience) or MoFlo Astrios EG (Beckman Coulter).

### Data analysis

All data were analysed with FlowJo v10 (FlowJo, LLC, Ashland, Oregon) and GraphPad Prism software (GraphPad Software; San Diego, California).

## Author Contributions

**Thomas R Müller:** Conceptualization; Formal analysis; Investigation; Methodology; Visualization; Writing‐original draft. **Corinna Schuler:** Investigation; Methodology; Writing‐review & editing. **Monika Hammel:** Methodology; Writing‐review & editing. **Amelie Köhler:** Methodology; Writing‐review & editing. **Sabrina Jutz:** Resources; Writing‐review & editing. **Judith Leitner:** Investigation; Methodology; Resources; Writing‐review & editing. **Kilian Schober:** Conceptualization; Methodology; Writing‐review & editing. **Dirk Busch:** Conceptualization; Formal analysis; Writing‐original draft. **Peter Steinberger:** Conceptualization; Methodology; Resources; Writing‐original draft.

## Conflict of interest

DHB is co‐founder of STAGE Cell Therapeutics GmbH (now Juno Therapeutics a Bristol‐Myers Squibb Company) and T Cell Factory B.V. (now Kite a Gilead Company). DHB has a consulting contract with and receives sponsored research support from Juno Therapeutics.

## References

[1] Roth TL , Puig‐Saus C , Yu R *et al* Reprogramming human T cell function and specificity with non‐viral genome targeting. Nature 2018; 559: 405–409. 29995861 10.1038/s41586-018-0326-5 PMC6239417

[2] Schober K , Müller TR , Gökmen F *et al* Orthotopic replacement of T‐cell receptor α‐ and β‐chains with preservation of near‐physiological T‐cell function. Nat Biomed Eng 2019; 3: 974–984. 31182835 10.1038/s41551-019-0409-0

[3] June CH , Riddell SR , Schumacher TN . Adoptive cellular therapy: a race to the finish line. Sci Transl Med 2015; 7: 280ps7. 10.1126/scitranslmed.aaa3643 25810311

[4] Guedan S , Ruella M , June CH . Emerging Cellular Therapies for Cancer. Annu Rev Immunol 2019; 37: 145–171. 30526160 10.1146/annurev-immunol-042718-041407 PMC7399614

[5] Morgan RA , Dudley ME , Wunderlich JR *et al* Cancer regression in patients after transfer of genetically engineered lymphocytes. Science 2006; 314: 126–129. 16946036 10.1126/science.1129003 PMC2267026

[6] Rapoport AP , Stadtmauer EA , Binder‐Scholl GK *et al* NY‐ESO‐1‐specific TCR‐engineered T cells mediate sustained antigen‐specific antitumor effects in myeloma. Nat Med 2015; 21: 914–921. 26193344 10.1038/nm.3910 PMC4529359

[7] Chodon T , Comin‐Anduix B , Chmielowski B *et al* Adoptive transfer of MART‐1 T‐cell receptor transgenic lymphocytes and dendritic cell vaccination in patients with metastatic melanoma. Clin Cancer Res 2014; 20: 2457–2465. 24634374 10.1158/1078-0432.CCR-13-3017 PMC4070853

[8] Linette GP , Stadtmauer EA , Maus MV *et al* Cardiovascular toxicity and titin cross‐reactivity of affinity‐enhanced T cells in myeloma and melanoma. Blood 2013; 122: 863–871. 23770775 10.1182/blood-2013-03-490565 PMC3743463

[9] Robbins PF , Morgan RA , Feldman SA *et al* Tumor regression in patients with metastatic synovial cell sarcoma and melanoma using genetically engineered lymphocytes reactive with NY‐ESO‐1. J Clin Oncol 2011; 29: 917–924. 21282551 10.1200/JCO.2010.32.2537 PMC3068063

[10] Shugay M , Britanova OV , Merzlyak EM *et al* Towards error‐free profiling of immune repertoires. Nat Methods 2014; 11: 653–655. 24793455 10.1038/nmeth.2960

[11] Mamedov IZ , Britanova OV , Zvyagin IV *et al* Preparing unbiased T‐cell receptor and antibody cDNA libraries for the deep next generation sequencing profiling. Front Immunol 2013; 4: 456. 24391640 10.3389/fimmu.2013.00456 PMC3870325

[12] Linnemann C , Heemskerk B , Kvistborg P *et al* High‐throughput identification of antigen‐specific TCRs by TCR gene capture. Nat Med 2013; 19: 1534–1541. 24121928 10.1038/nm.3359

[13] Spindler MJ , Nelson AL , Wagner EK *et al* Massively parallel interrogation and mining of natively paired human TCRαβ repertoires. Nat Biotechnol 2020; 38: 609–619. 32393905 10.1038/s41587-020-0438-y PMC7224336

[14] Dash P , Fiore‐Gartland AJ , Hertz T *et al* Quantifiable predictive features define epitope‐specific T cell receptor repertoires. Nature 2017; 547: 89–93. 28636592 10.1038/nature22383 PMC5616171

[15] Glanville J , Huang H , Nau A *et al* Identifying specificity groups in the T cell receptor repertoire. Nature 2017; 547: 94–98. 28636589 10.1038/nature22976 PMC5794212

[16] Peggs K , Verfuerth S , Pizzey A , Ainsworth J , Moss P , Mackinnon S . Characterization of human cytomegalovirus peptide‐specific CD8^+^ T‐cell repertoire diversity following *in vitro* restimulation by antigen‐pulsed dendritic cells. Blood 2002; 99: 213–223. 11756174 10.1182/blood.v99.1.213

[17] Trautmann L , Rimbert M , Echasserieau K *et al* Selection of T cell clones expressing high‐affinity public TCRs within human cytomegalovirus‐specific CD8 T cell responses. J Immunol 2005; 175: 6123–6132. 16237109 10.4049/jimmunol.175.9.6123

[18] Schub A , Schuster IG , Hammerschmidt W , Moosmann A . CMV‐specific TCR‐transgenic T cells for immunotherapy. J Immunol 2009; 183: 6819–6830. 19864595 10.4049/jimmunol.0902233

[19] Scheper W , Kelderman S , Fanchi LF *et al* Low and variable tumor reactivity of the intratumoral TCR repertoire in human cancers. Nat Med 2019; 25: 89–94. 30510250 10.1038/s41591-018-0266-5

[20] Dembić Z , Haas W , Weiss S *et al* Transfer of specificity by murine α and β T‐cell receptor genes. Nature 1986; 320: 232–238. 2421164 10.1038/320232a0

[21] Abraham RT , Weiss A . Jurkat T cells and development of the T‐cell receptor signalling paradigm. Nat Rev Immunol 2004; 4: 301–308. 15057788 10.1038/nri1330

[22] Jutz S , Leitner J , Schmetterer K *et al* Assessment of costimulation and coinhibition in a triple parameter T cell reporter line: Simultaneous measurement of NF‐κB, NFAT and AP‐1. J Immunol Methods 2016; 430: 10–20. 26780292 10.1016/j.jim.2016.01.007

[23] Dufva O , Koski J , Maliniemi P *et al* Integrated drug profiling and CRISPR screening identify essential pathways for CAR T‐cell cytotoxicity. Blood 2020; 135: 597–609. 31830245 10.1182/blood.2019002121 PMC7098811

[24] De Sousa Linhares A , Kellner F , Jutz S *et al* TIM‐3 and CEACAM1 do not interact in cis and in trans. Eur J Immunol 2020; 50: 1126–1141. 32222966 10.1002/eji.201948400 PMC7496933

[25] Rydzek J , Nerreter T , Peng H *et al* Chimeric Antigen Receptor Library Screening Using a Novel NF‐κB/NFAT Reporter Cell Platform. Mol Ther 2019; 27: 287–299. 30573301 10.1016/j.ymthe.2018.11.015 PMC6369451

[26] Renard V , Romero P , Vivier E , Malissen B , Luescher IF . CD8β increases CD8 coreceptor function and participation in TCR‐ligand binding. J Exp Med 1996; 184: 2439–2444. 8976201 10.1084/jem.184.6.2439 PMC2196369

[27] Wooldridge L , van den Berg HA , Glick M *et al* Interaction between the CD8 coreceptor and major histocompatibility complex class I stabilizes T cell receptor‐antigen complexes at the cell surface. J Biol Chem 2005; 280: 27491–27501. 15837791 10.1074/jbc.M500555200 PMC2441837

[28] Purbhoo MA , Boulter JM , Price DA *et al* The human CD8 coreceptor effects cytotoxic T cell activation and antigen sensitivity primarily by mediating complete phosphorylation of the T cell receptor zeta chain. J Biol Chem 2001; 276: 32786–32792. 11438524 10.1074/jbc.M102498200

[29] Viola A , Lanzavecchia A . T cell activation determined by T cell receptor number and tunable thresholds. Science 1996; 273: 104–106. 8658175 10.1126/science.273.5271.104

[30] van Loenen MM , de Boer R , Hagedoorn RS , van Egmond EHM , Falkenburg JHF , Heemskerk MHM . Optimization of the HA‐1‐specific T‐cell receptor for gene therapy of hematologic malignancies. Haematologica 2011; 96: 477–481. 21109688 10.3324/haematol.2010.025916 PMC3046283

[31] Okamoto S , Mineno J , Ikeda H *et al* Improved expression and reactivity of transduced tumor‐specific TCRs in human lymphocytes by specific silencing of endogenous TCR. Cancer Res 2009; 69: 9003–9011. 19903853 10.1158/0008-5472.CAN-09-1450

[32] Ahmadi M , King JW , Xue S‐A *et al* CD3 limits the efficacy of TCR gene therapy *in viv*o. Blood 2011; 118: 3528–3537. 21750319 10.1182/blood-2011-04-346338

[33] Provasi E , Genovese P , Lombardo A *et al* Editing T cell specificity towards leukemia by zinc finger nucleases and lentiviral gene transfer. Nat Med 2012; 18: 807–815. 22466705 10.1038/nm.2700 PMC5019824

[34] van Loenen MM , de Boer R , Amir AL *et al* Mixed T cell receptor dimers harbor potentially harmful neoreactivity. Proc Natl Acad Sci USA 2010; 107: 10972–10977. 20534461 10.1073/pnas.1005802107 PMC2890759

[35] Shan X , Czar MJ , Bunnell SC *et al* Deficiency of PTEN in Jurkat T cells causes constitutive localization of Itk to the plasma membrane and hyperresponsiveness to CD3 stimulation. Mol Cell Biol 2000; 20: 6945–6957. 10958690 10.1128/mcb.20.18.6945-6957.2000 PMC88770

[36] Hombrink P , Raz Y , Kester MGD *et al* Mixed functional characteristics correlating with TCR‐ligand koff ‐rate of MHC‐tetramer reactive T cells within the naive T‐cell repertoire. Eur J Immunol 2013; 43: 3038–3050. 23893393 10.1002/eji.201343397

[37] Derby MA , Wang J , Margulies DH , Berzofsky JA . Two intermediate‐avidity cytotoxic T lymphocyte clones with a disparity between functional avidity and MHC tetramer staining. Int Immunol 2001; 13: 817–824. 11369710 10.1093/intimm/13.6.817

[38] Laugel B , van den Berg HA , Gostick E *et al* Different T cell receptor affinity thresholds and CD8 coreceptor dependence govern cytotoxic T lymphocyte activation and tetramer binding properties. J Biol Chem 2007; 282: 23799–23810. 17540778 10.1074/jbc.M700976200

[39] Williams CM , Schonnesen AA , Zhang S‐Q *et al* Normalized Synergy Predicts That CD8 Co‐Receptor Contribution to T Cell Receptor (TCR) and pMHC Binding Decreases As TCR Affinity Increases in Human Viral‐Specific T Cells. Front Immunol 2017; 8: 894. 28804489 10.3389/fimmu.2017.00894 PMC5532383

[40] van Loenen MM , Hagedoorn RS , de Boer R , Falkenburg JHF , Heemskerk MHM . Extracellular Domains of CD8α and CD8ß Subunits Are Sufficient for HLA Class I Restricted Helper Functions of TCR‐Engineered CD4^+^ T Cells. PLoS One 2013; 8: e65212. 23738014 10.1371/journal.pone.0065212 PMC3667802

[41] Cohen CJ , Li YF , El‐Gamil M , Robbins PF , Rosenberg SA , Morgan RA . Enhanced antitumor activity of T cells engineered to express T‐cell receptors with a second disulfide bond. Cancer Res 2007; 67: 3898–3903. 17440104 10.1158/0008-5472.CAN-06-3986 PMC2147081

[42] Altman JD , Moss PA , Goulder PJ *et al* Phenotypic analysis of antigen‐specific T lymphocytes. Science 1996; 274: 94–96. 21690331

[43] Rosskopf S , Leitner J , Paster W *et al* A Jurkat 76 based triple parameter reporter system to evaluate TCR functions and adoptive T cell strategies. Oncotarget 2018; 9: 17608–17619. 29707134 10.18632/oncotarget.24807 PMC5915142

[44] Schaft N , Lankiewicz B , Gratama JW , Bolhuis RLH , Debets R . Flexible and sensitive method to functionally validate tumor‐specific receptors via activation of NFAT. J Immunol Methods 2003; 280: 13–24. 12972184 10.1016/s0022-1759(03)00067-x

[45] Mann SE , Zhou Z , Landry LG *et al* Multiplex T Cell Stimulation Assay Utilizing a T Cell Activation Reporter‐Based Detection System. Front Immunol 2020; 11: 633. 32328071 10.3389/fimmu.2020.00633 PMC7160884

[46] Zong S , Mi T , Flores LG *et al* Very rapid cloning, expression and identifying specificity of T‐cell receptors for T‐cell engineering. PLoS One 2020; 15: e0228112. 32040512 10.1371/journal.pone.0228112 PMC7010234

[47] Rosskopf S , Jutz S , Neunkirchner A *et al* Creation of an engineered APC system to explore and optimize the presentation of immunodominant peptides of major allergens. Sci Rep 2016; 6: 31580. 27539532 10.1038/srep31580 PMC4990899

[48] Derby M , Alexander‐Miller M , Tse R , Berzofsky J . High‐avidity CTL exploit two complementary mechanisms to provide better protection against viral infection than low‐avidity CTL. J Immunol 2001; 166: 1690–1697. 11160212 10.4049/jimmunol.166.3.1690

[49] Holler PD , Kranz DM . Quantitative analysis of the contribution of TCR/pepMHC affinity and CD8 to T cell activation. Immunity 2003; 18: 255–264. 12594952 10.1016/s1074-7613(03)00019-0

[50] Guo XJ , Dash P , Calverley M , Tomchuck S , Dallas MH , Thomas PG . Rapid cloning, expression, and functional characterization of paired αβ and γδ T‐cell receptor chains from single‐cell analysis. Mol Ther Methods Clin Dev 2016; 3: 15054. 26858965 10.1038/mtm.2015.54 PMC4729322

[51] Fischer DS , Wu Y , Schubert B , Theis FJ . Predicting antigen specificity of single T cells based on TCR CDR3 regions. Mol Syst Biol 2020; 16: e9416. 32779888 10.15252/msb.20199416 PMC7418512

[52] Morris EC , Tsallios A , Bendle GM , Xue S‐A , Stauss HJ . A critical role of T cell antigen receptor‐transduced MHC class I‐restricted helper T cells in tumor protection. Proc Natl Acad Sci USA 2005; 102: 7934–7939. 15908507 10.1073/pnas.0500357102 PMC1142362

[53] Xue S‐A , Gao L , Ahmadi M *et al* Human MHC Class I‐restricted high avidity CD4^+^ T cells generated by co‐transfer of TCR and CD8 mediate efficient tumor rejection *in vivo* . Oncoimmunology 2013; 2: e22590. 23483821 10.4161/onci.22590 PMC3583927

[54] Tan MP , Gerry AB , Brewer JE *et al* T cell receptor binding affinity governs the functional profile of cancer‐specific CD8^+^ T cells. Clin Exp Immunol 2015; 180: 255–270. 25496365 10.1111/cei.12570 PMC4408161

[55] Dössinger G , Bunse M , Bet J *et al* MHC Multimer‐Guided and Cell Culture‐Independent Isolation of Functional T Cell Receptors from Single Cells Facilitates TCR Identification for Immunotherapy. PLoS One 2013; 8: e61384. 23637823 10.1371/journal.pone.0061384 PMC3637308

[56] Nakagawa S , Niimura Y , Gojobori T , Tanaka H , Miura K . Diversity of preferred nucleotide sequences around the translation initiation codon in eukaryote genomes. Nucleic Acids Res 2008; 36: 861–871. 18086709 10.1093/nar/gkm1102 PMC2241899

[57] Cohen CJ , Zhao Y , Zheng Z , Rosenberg SA , Morgan RA . Enhanced antitumor activity of murine‐human hybrid T‐cell receptor (TCR) in human lymphocytes is associated with improved pairing and TCR/CD3 stability. Cancer Res 2006; 66: 8878–8886. 16951205 10.1158/0008-5472.CAN-06-1450 PMC2147082

[58] Kuball J , Dossett ML , Wolfl M *et al* Facilitating matched pairing and expression of TCR chains introduced into human T cells. Blood 2007; 109: 2331–2338. 17082316 10.1182/blood-2006-05-023069 PMC1852191

[59] Ren J , Liu X , Fang C , Jiang S , June CH , Zhao Y . Multiplex genome editing to generate universal CAR T cells resistant to PD1 inhibition. Clin Cancer Res 2017; 23: 2255–2266. 27815355 10.1158/1078-0432.CCR-16-1300 PMC5413401

[60] Effenberger M , Stengl A , Schober K *et al* FLEXamers: A Double Tag for Universal Generation of Versatile Peptide‐MHC Multimers. J Immunol 2019; 202: 2164–2171. 30760621 10.4049/jimmunol.1801435

