# Supplementary material for: A T‐cell reporter platform for high‐throughput and reliable investigation of TCR function and biology
Source: Clin Transl Immunology. 2020 Nov 23;9(11):e1216. doi: 10.1002/cti2.1216 (PMC7681835; doi:10.1002/cti2.1216)
Supplement: Supplementary file 1 [file CTI2-9-e1216-s001.pdf]

Supplementary Information

A T-cell reporter platform for high-throughput and reliable investigation of TCR function and biology

Thomas R. Müller, Corinna Schuler, Monika Hammel, Amelie Köhler, Sabrina Jutz, Judith Leitner, Kilian Schober, Dirk H. Busch & Peter Steinberger

Suppl. Table 1. Information on the TCRs used in this study

Virus	epitop	peptide	residue	HLA restrictio	donor	TCR id	TRBV	TRBD	TRBJ	CDR3β aa	TRAV	TRAJ	CDR3α aa	public
CMV	pp50	VTEHDTLLY	245-253	A*01:01	HZ225	TCR 14-11	TRBV5-4*00(885.8)	TRBD1*00(35)	TRBJ1-2*00(237.1)	CASSLRGAGNYGYTF	TRAV29DV5*00(862.3	TRAJ23*00(269.3)	CAAIDQGGKLIF	
CMV	pp50	VTEHDTLLY	245-253	A*01:01	HZ225	TCR 15-11	TRBV5-4*00(750.7)	TRBD1*00(45)	TRBJ1-2*00(274)	CASSLRGQGNYGYTF	TRAV29DV5*00(881.1	TRAJ23*00(320.1)	CAAINQGGKLIF	
CMV	pp50	VTEHDTLLY	245-253	A*01:01	HZ225	TCR 16-11	TRBV5-4*00(716.7)	TRBD1*00(50)	TRBJ1-2*00(271.6)	CASSTRGQGSYGYTF	TRAV29DV5*00(940.2	TRAJ23*00(308.9)	CAAINQGGKLIF	
CMV	pp50	VTEHDTLLY	245-253	A*01:01	HZ225	TCR 17-11	TRBV7-9*00(762.3)	TRBD2*00(27),TRBD1*00(25	TRBJ2-7*00(255.7)	CASSAEAGHPYEQYF	TRAV29DV5*00(887.7	TRAJ54*00(268.3)	CAARPGAQKLVF	
CMV	pp50	VTEHDTLLY	245-253	A*01:01	HZ225	TCR 18-11	TRBV9*00(620.3)	TRBD1*00(35)	TRBJ2-7*00(271.2)	CASSVGQGVVYEQYF	TRAV35*00(875.3)	TRAJ10*00(384.6)	CAGQAPGTILTGGGNKLTF	
CMV	pp50	VTEHDTLLY	245-253	A*01:01	HZ225	TCR 19-11	TRBV20-1*00(726.1)	TRBD2*00(45)	TRBJ2-3*00(251)	CSARRLAGPDPTDTQYF	TRAV19*00(528)	TRAJ37*00(352.4)	CALPVLGSSNTGKLIF	
CMV	pp50	VTEHDTLLY	245-253	A*01:01	HZ225	TCR 20-11	TRBV6-1*00(789.9)	TRBD1*00(55)	TRBJ1-5*00(231.6)	CASSATRGQGGQPQHF	TRAV19*00(586.8)	TRAJ17*00(413.7)	CALSETPLIKAAGNKLTF	
CMV	pp50	VTEHDTLLY	245-253	A*01:01	HZ225	TCR 21-11	TRBV9*00(680.2)	TRBD2*00(40)	TRBJ2-7*00(265.4)	CASSVYGGPTYEQYF	TRAV41*00(758)	TRAJ49*00(294.7)	CAVRYTFGNQFYF	
CMV	pp50	VTEHDTLLY	245-253	A*01:01	HZ225	TCR 22-11	TRBV24-1*00(859.2)	TRBD2*00(51)	TRBJ2-1*00(211.7)	CATSAPGLASQEQFF	TRAV41*00(803.1)	TRAJ49*00(283.8)	CAVYGNQFYF	
CMV	pp50	VTEHDTLLY	245-253	A*01:01	HZ225	TCR 23-11	TRBV9*00(478.1)	TRBD1*00(30)	TRBJ2-7*00(345.2)	CASSVGQGSSYEQYF	TRAV36DV7*00(922.5	TRAJ54*00(333)	CAVYSIQGAQKLVF	
CMV	pp50	VTEHDTLLY	245-253	A*01:01	HZ225	TCR 24-11	TRBV12-3*00(651.5),TRBV12-4*00(623.4)		TRBJ2-7*00(258.9)	CASSLAQSASYEQYF	TRAV12-1*00(902.1)	TRAJ3*00(313.6)	CVVSGSSASKIIF	
CMV	pp50	VTEHDTLLY	245-253	A*01:01	HZ1003	TCR 25-2	TRBV5-4*00(615.4)	TRBD1*00(45)	TRBJ1-2*00(276)	CASSSRGQGNYGYTF	TRAV29DV5*00(855.9	TRAJ23*00(298.1)	CAAINQGGKLIF	
CMV	pp50	VTEHDTLLY	245-253	A*01:01	HZ1003	TCR 26-2	TRBV7-9*00(563.9)	TRBD2*00(28),TRBD1*00(25	TRBJ2-1*00(243)	CASSLESSPTPWEQFF	TRAV29DV5*00(942.3	TRAJ54*00(293.6)	CAARPGAQKLVF	
CMV	pp50	VTEHDTLLY	245-253	A*01:01	HZ1003	TCR 48-4	TRBV12-3*00(615.7),TRBV12-4*00(563.5)		TRBJ1-2*00(256.8)	CASSFGGNTPYGYTF	TRAV13-2*00(892.4)	TRAJ12*00(321.8)	CAENNRLDSSYKLIF	
CMV	pp50	VTEHDTLLY	245-253	A*01:01	HZ1003	TCR 49-4	TRBV6-2*00(702.9),TRBV6-3*00(701.4)		TRBJ1-6*00(362.6)	CASKFPTGRSSYNSPLHF	TRAV21*00(848.1)	TRAJ49*00(320)	CAVPINTGNQFYF	
CMV	pp50	VTEHDTLLY	245-253	A*01:01	HZ603	TCR 60-4	TRBV7-9*01 F	TRBD2*01 F	TRBJ2-7*01 F	CASSLEGSSRPYEQYF	TRAV29/DV5*01 F	TRAJ44*01 F	CAARGTASKLTF	
CMV	pp50	VTEHDTLLY	245-253	A*01:01	HZ603	TCR 61-4	TRBV7-9*01 F, or TRBV7-9*02 (F) or TRBV7-9*03 F or TRBV7-9*04 (F) or TRBV7-9*05 (F)	TRBD2*02 F	TRBJ2-7*01 F	CASSLEFSGTPYEQYF	TRAV29/DV5*01 F	TRAJ34*01 F	CAARSGADKLIF	
CMV	pp50	VTEHDTLLY	245-253	A*01:01	HZ603	TCR 62-4	TRBV7-9*01 F, or TRBV7-9*02 (F) or TRBV7-9*0	TRBD2*01 F	TRBJ2-7*01 F	CASSLEYGSKPYEQYF	TRAV29/DV5*01 F	TRAJ31*01 F	CAARRNNARLMF	
CMV	pp50	VTEHDTLLY	245-253	A*01:01	HZ603	TCR 63-4	TRBV9*01 F	TRBD1*01 F	TRBJ2-7*01 F	CASSVESGPTYEQYF	TRAV41*01 F	TRAJ49*01 F	CAVRFTYGNQFYF	
CMV	pp65	NLVPMVATV	495-503	A*02:01	HZ510	TCR 1-4	TRBV27*01 F	TRBD2*02 F	TRBJ2-3*01 F	CASSLNVVAGVTDTQYF	TRAV8-3*01 F	TRAJ49*01 F	CAVGTGNQFYF	
CMV	pp65	NLVPMVATV	495-503	A*02:01	HZ510	TCR 2-4	TRBV24-1*01 F	TRBD1*01 F	TRBJ1-5*01 F	CATSDWDGTKGFRQPQH	TRAV5*01 F	TRAJ23*01 F	CAEMSNQGGKLIF	
CMV	pp65	NLVPMVATV	495-503	A*02:01	HZ510	TCR 3-4	TRBV4-3*01 F, or TRBV4-3*04 (F)	TRBD1*01 F	TRBJ1-2*01 F	CASRAGQAWDGYTF	TRAV24*01 F	TRAJ26*01 F	CARNYGQNFVF	
CMV	pp65	NLVPMVATV	495-503	A*02:01	HZ510	TCR 4-4	TRBV7-6*00(1526.7)	TRBD1*00(40),TRBD2*00(40	TRBJ1-4*00(562.7)	CASSLAPGATNEKLFF	TRAV26-2*00(1766.7)	TRAJ43*00(551.9)	CIVDNNNDMRF	yes
CMV	pp65	NLVPMVATV	495-503	A*02:01	HZ1016	TCR 5-2	TRBV6-5*01 F	TRBD1*01 F	TRBJ1-2*01 F	CASSYKTGTSYGYTF	TRAV24*01 F	TRAJ49*01 F	CARNSGNQFYF	
CMV	pp65	NLVPMVATV	495-503	A*02:01	HZ1016	TCR 6-2	TRBV12-4*00(1953.6),TRBV12-3*00(1644)		TRBJ1-2*00(519.9)	CASSSANYGYTF	TRAV35*00(2134.7)	TRAJ50*00(641.5)	CAGPMKTSYDKVIF	yes
CMV	pp65	NLVPMVATV	495-503	A*02:01	HZ961	TCR 10-4	TRBV28*01 F	TRBD1*01 F	TRBJ1-1*01 F	CASSFQGFTEAFF	TRAV3*01 F	TRAJ26*01 F	CAVYYGQNFVF	
CMV	pp65	NLVPMVATV	495-503	A*02:01	HZ961	TCR 11-4	TRBV28*01 F	TRBD1*01 F	TRBJ1-1*01 F	CASSFQGFTQAFF	TRAV3*01 F	TRAJ26*01 F	CAVYYGQNFVF	
CMV	pp65	NLVPMVATV	495-503	A*02:01	HZ561	TCR 50-10	TRBV24-1*00(1429.5)	TRBD1*00(30),TRBD2*00(30	TRBJ2-4*00(570.9)	CATSSSDLAKNIQYF	TRAV26-2*00(1485.1)	TRAJ45*00(570.8)	CILWGGADGLTF	
CMV	pp65	NLVPMVATV	495-503	A*02:01	HZ561	TCR 51-10	TRBV6-5*00(1289.9)	TRBD1*00(70)	TRBJ1-5*00(508.2)	CASSKTTGVRNQPQHF	TRAV24*00(1487.4)	TRAJ39*00(502.5)	CASPLGNMLTF	
CMV	pp65	NLVPMVATV	495-503	A*02:01	HZ561	TCR 52-10	TRBV13*00(1322.4)	TRBD1*00(90)	TRBJ1-1*00(439.4)	CASSLGGQGVSEAFF	TRAV5*00(1320.4)	TRAJ36*00(627.1)	CAERLQTGANNLFF	
CMV	pp65	NLVPMVATV	495-503	A*02:01	HZ561	TCR 53-10	TRBV7-8*00(1513.7)	TRBD2*00(40)	TRBJ2-5*00(455.5)	CASSLSLLWSTVGETQYF	TRAV21*00(1904.8)	TRAJ27*00(588.1)	CAVNTNAGKSTF	
CMV	pp65	NLVPMVATV	495-503	A*02:01	HZ561	TCR 54-10	TRBV27*00(1664.9)	TRBD1*00(50),TRBD2*00(50	TRBJ2-1*00(455.4)	CASSLSVAGENNEQFF	TRAV24*00(1650.4)	TRAJ21*00(522.1)	CAFNSFNKFYF	
CMV	pp65	NLVPMVATV	495-503	A*02:01	HZ561	TCR 55-10	TRBV15*00(1333)	TRBD1*00(70)	TRBJ2-6*00(611.2)	CATSIWDRSSGANVLTF	TRAV41*00(1751.1)	TRAJ48*00(613.2)	CAVRGNFGNEKLTF	
CMV	pp65	NLVPMVATV	495-503	A*02:01	HZ561	TCR 57-10	TRBV24-1*00(1132.1)	TRBD2*00(150)	TRBJ2-5*00(488.5)	CATSPGTSGAYQETQYF	TRAV26-2*00(1184)	TRAJ32*00(596.2)	CILRDGGGATNKLIF	
CMV	pp65	NLVPMVATV	495-503	A*02:01	HZ612	TCR 65-8	TRBV4-2*00(1582.7),TRBV4-3*00(1307.6)	TRBD1*00(70),TRBD2*00(70	TRBJ2-5*00(453.9)	CASSQDGGGGLETQYF	TRAV24*00(1211.4)	TRAJ40*00(609.8)	CAFRGTSGTYKYIF	
CMV	pp65	NLVPMVATV	495-503	A*02:01	HZ612	TCR 66-8	TRBV6-5*00(1014.7)	TRBD1*00(40),TRBD2*00(40	TRBJ2-1*00(491.8)	CASRSHHNEQFF	TRAV24*00(1005.4)	TRAJ11*00(492.4)	CAFIDSTLTF	
CMV	pp65	NLVPMVATV	495-503	A*02:01	HZ612	TCR 68-8	TRBV7-3*00(1282.9)	TRBD2*00(40)	TRBJ2-6*00(628.7)	CASSLIAMGSGANVLTF	TRAV8-3*00(951)	TRAJ37*00(640.4)	CAGGGSSNTGKLIF	
CMV	pp65	NLVPMVATV	495-503	A*02:01	HZ612	TCR 69-8	TRBV6-5*00(1423.5)	TRBD1*00(110)	TRBJ1-2*00(461.2)	CASSPVTGTGFYGYTF	TRAV24*00(1565.5)	TRAJ49*00(578.6)	CALNTGNQFYF	yes
CMV	pp65	NLVPMVATV	495-503	A*02:01	HZ633	TCR 72-14	TRBV27*00(867.6)	TRBD1*00(70)	TRBJ1-2*00(440.2)	CASSLAQGTAYLGYTF	TRAV8-3*00(616)	TRAJ26*00(597.7)	CAVANGQNFVF	
CMV	pp65	NLVPMVATV	495-503	A*02:01	HZ633	TCR 73-14	TRBV13*00(761.1)	TRBD1*00(100)	TRBJ1-1*00(438.2)	CASSSGGQGVSRAFF	TRAV5*00(1159.5)	TRAJ36*00(636.1)	CAERLQTGANNLFF	
CMV	pp65	NLVPMVATV	495-503	A*02:01	HZ633	TCR 74-14	TRBV13*00(1802.6)	TRBD1*00(60)	TRBJ2-7*00(447.9)	CASSFGGGGYEQYF	TRAV8-6*00(1699)	TRAJ48*00(506.2)	CAVSGLNEKLTF	
CMV	pp65	NLVPMVATV	495-503	A*02:01	HZ633	TCR 75-14	TRBV27*00(1567.4)	TRBD1*00(50)	TRBJ2-1*00(491.8)	CASSSVGGSPYNEQFF	TRAV3*00(1726.7)	TRAJ35*00(542.4)	CAVAFGNVLHC	
CMV	pp65	NLVPMVATV	495-503	A*02:01	HZ633	TCR 76-14	TRBV30*00(1351.4)	TRBD1*00(30),TRBD2*00(30	TRBJ2-4*00(569.4)	CAWSISDLAKNIQYF	TRAV26-2*00(1859.2)	TRAJ43*00(517.5)	CILSDNNDMRF	
CMV	pp65	NLVPMVATV	495-503	A*02:01	HZ633	TCR 77-14	TRBV6-5*00(965.6)	TRBD1*00(80)	TRBJ1-2*00(513.1)	CASSYQTGASYGYTF	TRAV24*00(1050)	TRAJ49*00(597)	CARNTGNQFYF	yes
CMV	pp65	NLVPMVATV	495-503	A*02:01	HZ633	TCR 78-14	TRBV6-5*00(1403.8)	TRBD1*00(100)	TRBJ1-2*00(465.9)	CASSPITGTGFYGYTF	TRAV24*00(1431.1)	TRAJ49*00(590)	CAQNTGNQFYF	
CMV	pp65	NLVPMVATV	495-503	A*02:01	HZ633	TCR 80-14	TRBV6-2*00(534.2),TRBV6-3*00(529.3)	TRBD1*00(40)	TRBJ1-1*00(538.2)	CASSYFGVNTEAFF	TRAV17*00(412.3)	TRAJ26*00(581.1)	CATDGDYGQNFVF	
CMV	pp65	NLVPMVATV	495-503	A*02:01	HZ633	TCR 81-14	TRBV20-1*00(1904.2)	TRBD1*00(60),TRBD2*00(60	TRBJ2-3*00(478)	CSAPVGGGGFTDTQYF	TRAV5*00(1661.5)	TRAJ23*00(541.2)	CAERSEGQGGKLIF	
CMV	pp65	NLVPMVATV	495-503	A*02:01	HZ633	TCR 82-14	TRBV7-6*00(1310.7)	TRBD1*00(60),TRBD2*00(60	TRBJ1-4*00(569.2)	CASSLAPGATNEKLFF	TRAV26-2*00(1637.6)	TRAJ43*00(539.9)	CILDNNNDMRF	yes
CMV	pp65	NLVPMVATV	495-503	A*02:01	HZ656	TCR 83-3	TRBV29-1*00(1457.2)	TRBD1*00(90)	TRBJ2-7*00(456.5)	CSVAGTVNEQYF	TRAV24*00(2357.9)	TRAJ57*00(641.5)	CAFETQGGSEKLVF	
CMV	pp65	NLVPMVATV	495-503	A*02:01	HZ656	TCR 84-3	TRBV6-5*00(1716.1)	TRBD1*00(100)	TRBJ1-2*00(465.2)	CASSPISGTGSYGYTF	TRAV24*00(1615.2)	TRAJ49*00(579.8)	CARNTGNQFYF	
CMV	pp65	NLVPMVATV	495-503	A*02:01	HZ963	TCR 85-1	TRBV27*00(1853)	TRBD2*00(50)	TRBJ2-1*00(480.1)	CASSPVAGALYNEQFF	TRAV8-3*00(1722.1)	TRAJ49*00(530.1)	CAVAHGNQFYF	

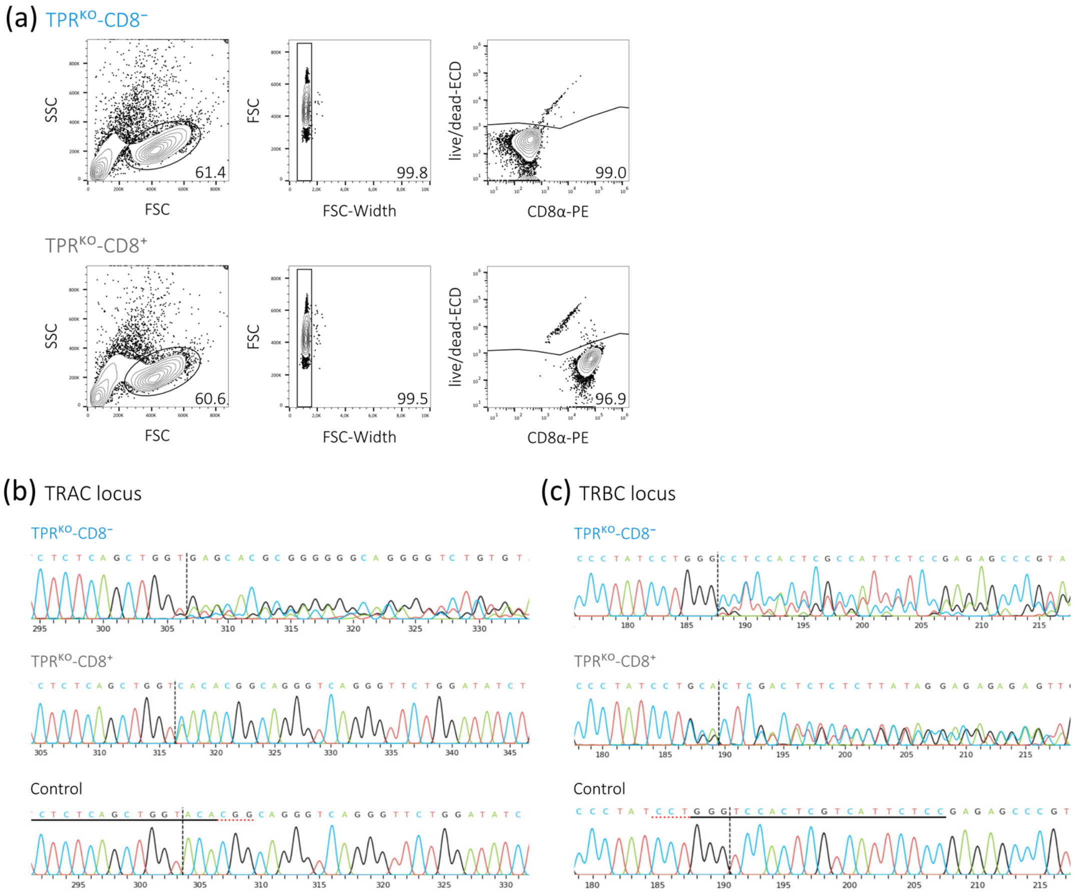

Suppl. Fig. 1. Generation of CD8$^{+/-}$ TCR-replaced Jurkat triple parameter reporter T-cell lines. (a) Gating strategy of flow cytometry-sorting of CD3-negative cells after CRISPR/Cas9-mediated TCR-KO (shown in Fig. 1a). **(b)** Sequence analysis of TCR α-chain locus (TRAC). Vertical dashed line indicates intended cut site. Three indel-events in TPR^{KO}-CD8^{-} and one indel-event in TPR^{KO}-CD8^{+} were detected, all resulting in frameshifts. **(c)** Sequence analysis of TCR β-chain locus (TRBC). Vertical dashed line indicates intended cut site. Three indel-events in TPR^{KO}-CD8^{-} and three indel-events in TPR^{KO}-CD8^{+} were detected, all resulting in frameshifts.

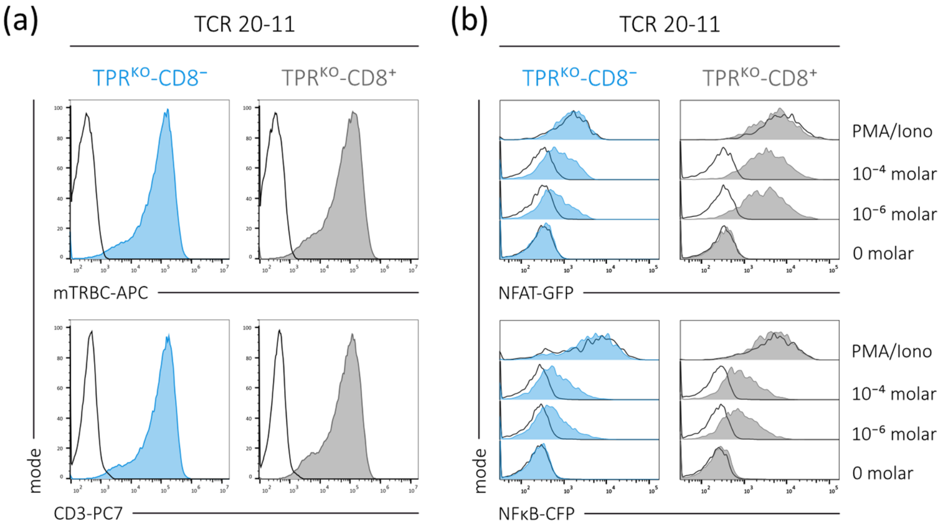

Suppl. Fig. 2. Surface expression and function of transgenically expressed TCR20-11 in TPR^{KO} cell lines. (a) Retroviral transduction of TPR^{KO}-CD8^{-} (blue) and TPR^{KO}-CD8^{+} (grey) cells with an A1/pp50-specific TCR containing murine constant TCR α/β-chains. mTRBC staining and re-expression of CD3 indicate expression of transgenic TCR. Black line represents TCR-untransduced mock control. **(b)** NFAT and NFκB reporter signal after 24h of stimulation of TCR20-11 expressing TPR^{KO}-CD8^{-} and TPR^{KO}-CD8^{+} cells either with PMA/Iono or A1/pp50 peptide-pulsed HLA-A*0101-positive K562 at indicated concentrations. Black line represents TCR-untransduced mock control.

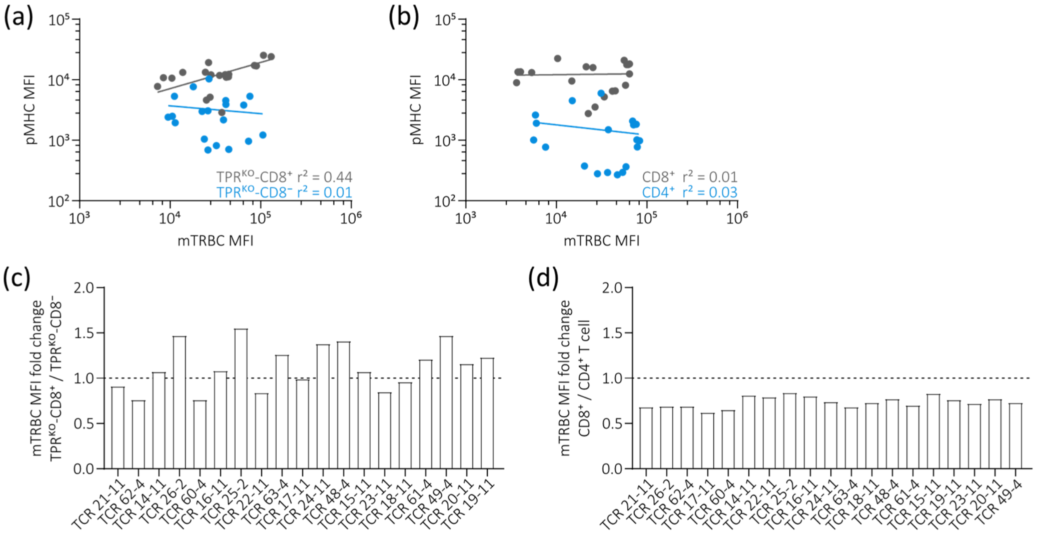

Suppl. Fig. 3. pMHC-multimer staining in TPR^{KO} cell lines and primary T cells is a TCR-intrinsic feature and not directly linked to TCR surface expression. (a/b) Correlation of transgenic TCR surface expression (indicated by mTRBC MFI) and pMHC-multimer staining in TPR^{KO} cell lines (a) and endogenous TCR-KO primary T cells (b). Each dot represents one of 19 individual TCRs. Fitting by non-linear regression. **(c/d)** Quantification of CD8αβ co-receptor dependency (calculated by fold change of TPR^{KO}-CD8^{+}/TPR^{KO}-CD8^{-} or CD8^{+}/CD4^{+} respectively) of TCR surface expression for 19 individual A1/pp50-specific transgenically expressed TCRs in TPR^{KO} cell lines (c) and endogenous TCR-KO primary T cells (d). TCRs are ordered from left to right according to CD8αβ co-receptor dependency of pMHC-multimer staining shown in Fig. 2g/h. Dashed line represents no change.

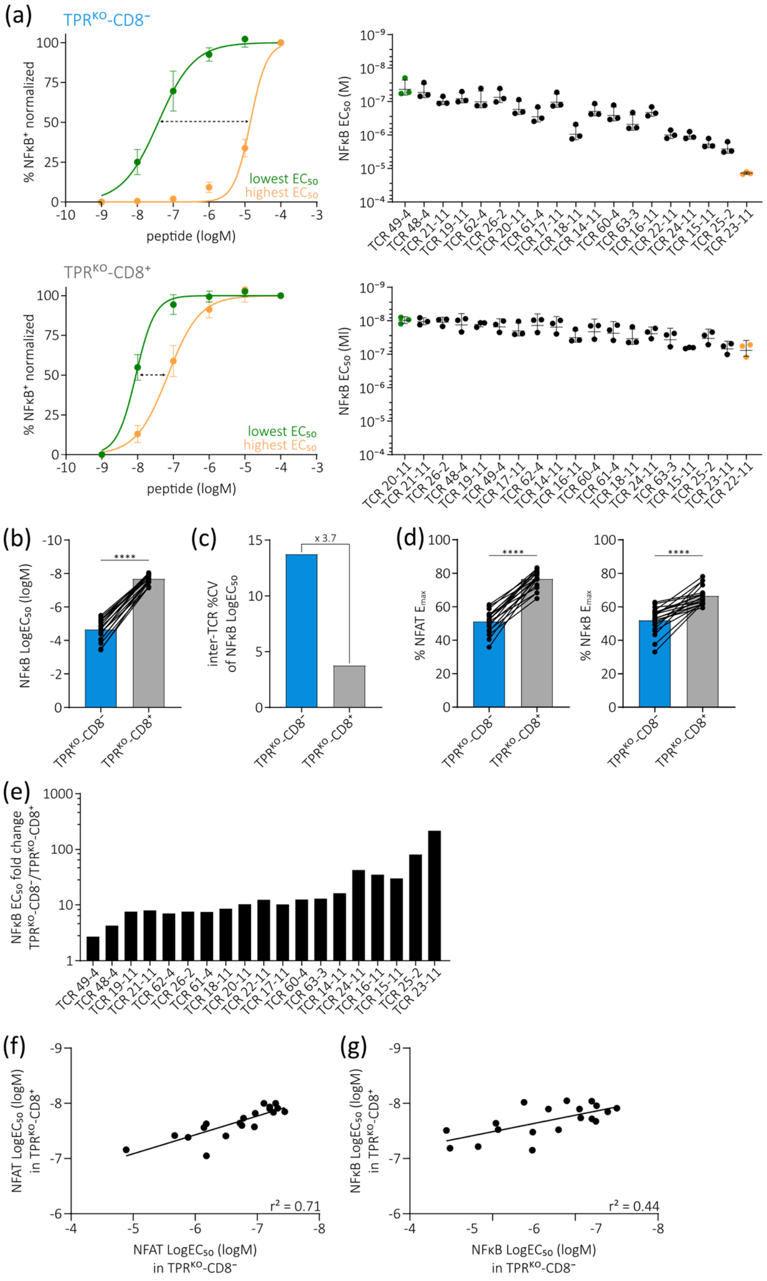

Suppl. Fig. 4. TPR^{KO} cell lines facilitate high resolution assessment of TCR functionality. (a) NFκB reporter EC_{50} curves of most (lowest EC_{50}, green) and least (highest EC_{50}, orange) antigen-sensitive TCRs (left) and quantification of EC_{50} of 19 A1/pp50-specific transgenically expressed TCRs in TPR^{KO} cell lines (right). Stimulation assay as in Fig. 3a. TCRs are ordered from left to right according to NFAT EC_{50} shown in Fig. 3b. Depicted are replicates and mean ± s.d.. **(b)** Comparison of NFκB reporter LogEC_{50} of 19 A1/pp50-specific TCRs in TPR^{KO} cell lines with (grey) and without (blue) CD8αβ co-receptor. Each dot represents one of 19 individual TCRs. Statistical testing by two-tailed paired Student's *t*-test, ****$P < 0.0001$. **(c)** Quantification of NFκB LogEC_{50} variability between 19 A1/pp50 TCRs. Fold change between TPR^{KO} cell lines is indicated. **(d)** Quantification of NFAT (left) and NFκB (right) maximal reporter response (as percentage reporter$^+$ of whole population) in TPR^{KO} cell lines. Each dot represents one of 19 individual TCRs. Statistical testing by two-tailed paired Student's *t*-test, ****$P < 0.0001$. **(e)** Quantification of CD8αβ co-receptor dependency (calculated by fold change of TPR^{KO}-CD8$^-$/TPR^{KO}-CD8$^+$; high value represents high dependency) of NFκB reporter EC_{50} for 19 individual A1/pp50-specific transgenically expressed TCRs in TPR^{KO} cell lines. TCRs are ordered from left to right according to CD8αβ co-receptor dependency of NFAT EC_{50} shown in Fig. 3f. Each bar represents the mean of three replicates. **(f/g)** Correlation of NFAT reporter LogEC_{50} (f) and NFκB reporter LogEC_{50} (g) of 19 A1/pp50-specific transgenically expressed TCRs in TPR^{KO} cell lines with and without CD8αβ co-receptor. Each dot represents one of 19 individual TCRs. Fitting by linear regression.

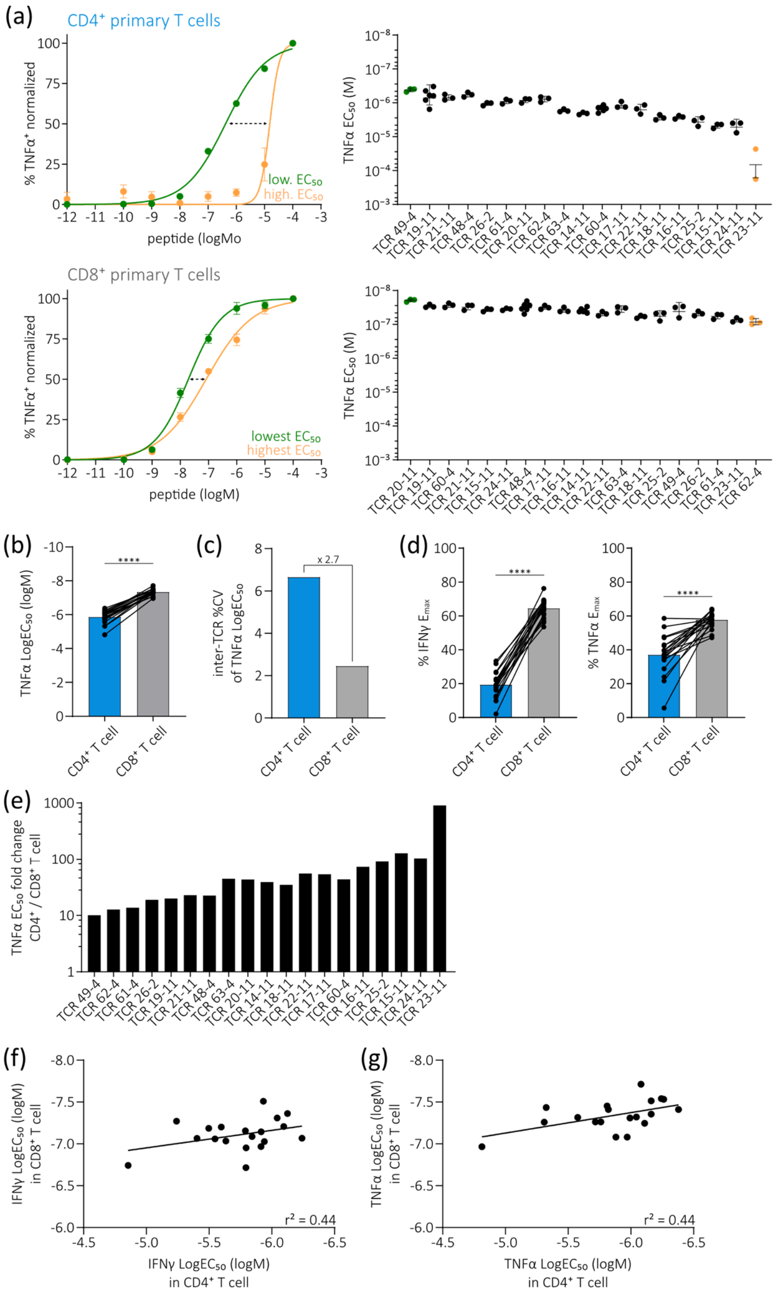

Suppl. Fig. 5. Determination of TCR functionality in CD4+/CD8+ primary T cells. (a) TNFα EC_{50} curves of most (lowest EC_{50}, green) and least (highest EC_{50}, orange) antigen-sensitive TCRs (left) and quantification of EC_{50} of 19 A1/pp50-specific transgenically expressed TCRs in endogenous TCR-KO primary T cells. Stimulation assay as in Fig. 4a. TCRs are ordered from left to right according to IFNγ EC_{50} shown in Fig. 4b. Depicted are replicates and mean ± s.d.. **(b)** Comparison of TNFα $LogEC_{50}$ of 19 A1/pp50-specific TCRs in CD4+ (blue) and CD8+ (grey) endogenous TCR-KO primary T cells. Each dot represents one of 19 individual TCRs. Statistical testing by two-tailed paired Student's *t*-test, $^{****}P < 0.0001$. **(c)** Quantification of TNFα $LogEC_{50}$ variability between 19 A1/pp50 TCRs. Fold change between CD4+ and CD8+ T cells is indicated. **(d)** Quantification of IFNγ (left) and TNFα (right) maximal reporter response (as percentage cytokine+ of whole population) in endogenous TCR-KO primary T cells. Each dot represents one of 19 individual A1/pp50-specific TCRs. Statistical testing by two-tailed paired Student's *t*-test, $^{****}P < 0.0001$. **(e)** Quantification of CD8αβ co-receptor dependency (calculated by fold change of CD4+/CD8+; high value represents high dependency) of TNFα EC_{50} for 19 individual A1/pp50-specific transgenically expressed in endogenous TCR-KO primary T cells. TCRs are ordered from left to right according to CD8αβ co-receptor dependency of IFNγ EC_{50} shown in Fig. 4f. Each bar represents the mean of three replicates. **(f/g)** Comparison of IFNγ $LogEC_{50}$ (f) and TNFα $LogEC_{50}$ (g) of 19 A1/pp50-specific TCRs in CD4+ and CD8+ endogenous TCR-KO primary T cells. Each dot represents one of 19 individual TCRs. Fitting by linear regression.

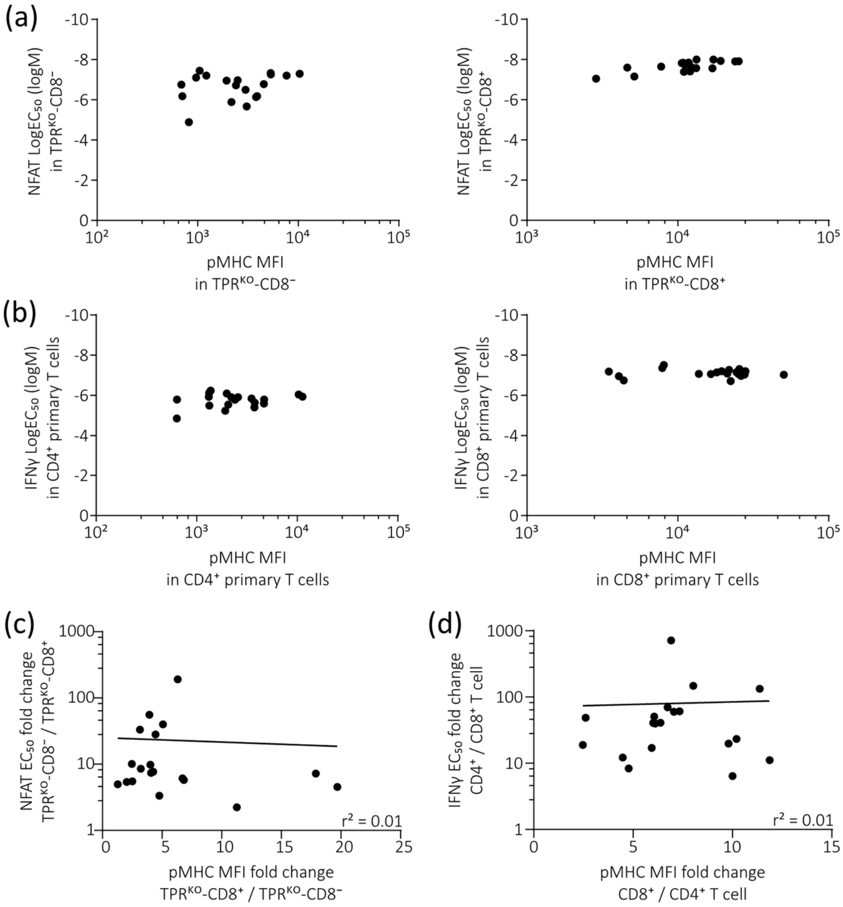

Suppl. Fig. 6. pMHC-multimer staining does not correlate to functional avidity in TPR^{KO} cell lines and primary T cells. **(a)** Correlation of pMHC-multimer staining to NFAT reporter $LogEC_{50}$ in TPR^{KO}-$CD8^-$ (left) and TPR^{KO}-$CD8^+$ (right). **(b)** Correlation of pMHC multimer staining to IFNγ $LogEC_{50}$ in $CD4^+$ (left) and $CD8^+$ (right) endogenous TCR-KO primary T cells. **(c)** Correlation of CD8αβ co-receptor dependency of pMHC-multimer staining and CD8αβ co-receptor dependency of NFAT reporter $LogEC_{50}$ in TPR^{KO} cell lines. **(d)** Correlation of CD8αβ co-receptor dependency of pMHC-multimer staining and CD8αβ co-receptor dependency of IFNγ $LogEC_{50}$ in endogenous TCR-KO primary T cells. In all graphs, each dot represents one of 19 individual A1/pp50-specific transgenically expressed TCRs. If possible, fitting by non-linear regression.

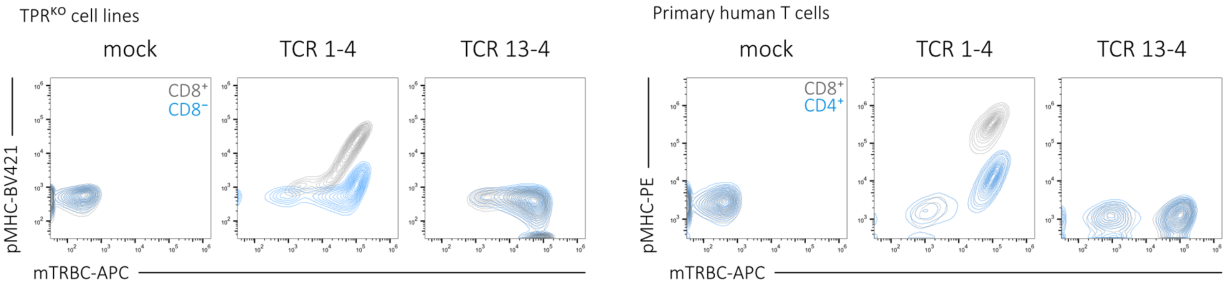

Suppl. Fig. 7. Transgenic re-expression of TCRs in TPR^{KO}-CD8^{+} cell line and pMHC-multimer staining facilitate validation of antigen-HLA specificity. Flow cytometry mTRBC and A2/pp65 pMHC-multimer co-staining of two transgenically expressed TCRs in TPR^{KO} cell lines (left) and endogenous TCR-KO primary T cells (right).

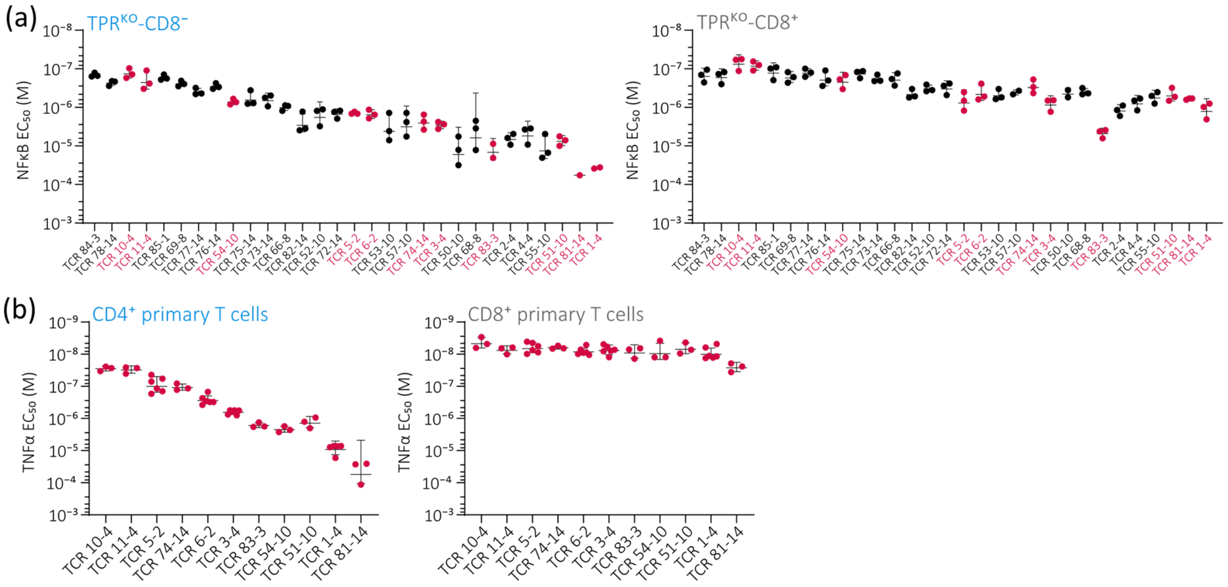

Suppl. Fig. 8. TPR^{KO} cell lines as the centerpiece of a high-throughput TCR screening platform. (a) Quantification of NFκB reporter EC_{50} of 30 A2/pp65-specific TCRs in TPR^{KO}-CD8^{-} (left) and TPR^{KO}-CD8^{+} (right). Eleven TCRs marked in red were selected for further functional testing in primary T cells. TCRs are ordered from left to right according to NFAT EC_{50} in TPR^{KO}-CD8^{-} shown in Fig. 6c. Depicted are replicates and mean ± s.d.. **(b)** Quantification of TNFα EC_{50} of eleven selected A2/pp65-specific TCRs in CD4^{+} (left) and CD8^{+} (right) endogenous TCR-KO primary T cells. TCRs are ordered from left to right according to IFNγ EC_{50} in CD4^{+} primary T cells shown in Fig. 6d. Depicted are replicates and mean ± s.d..

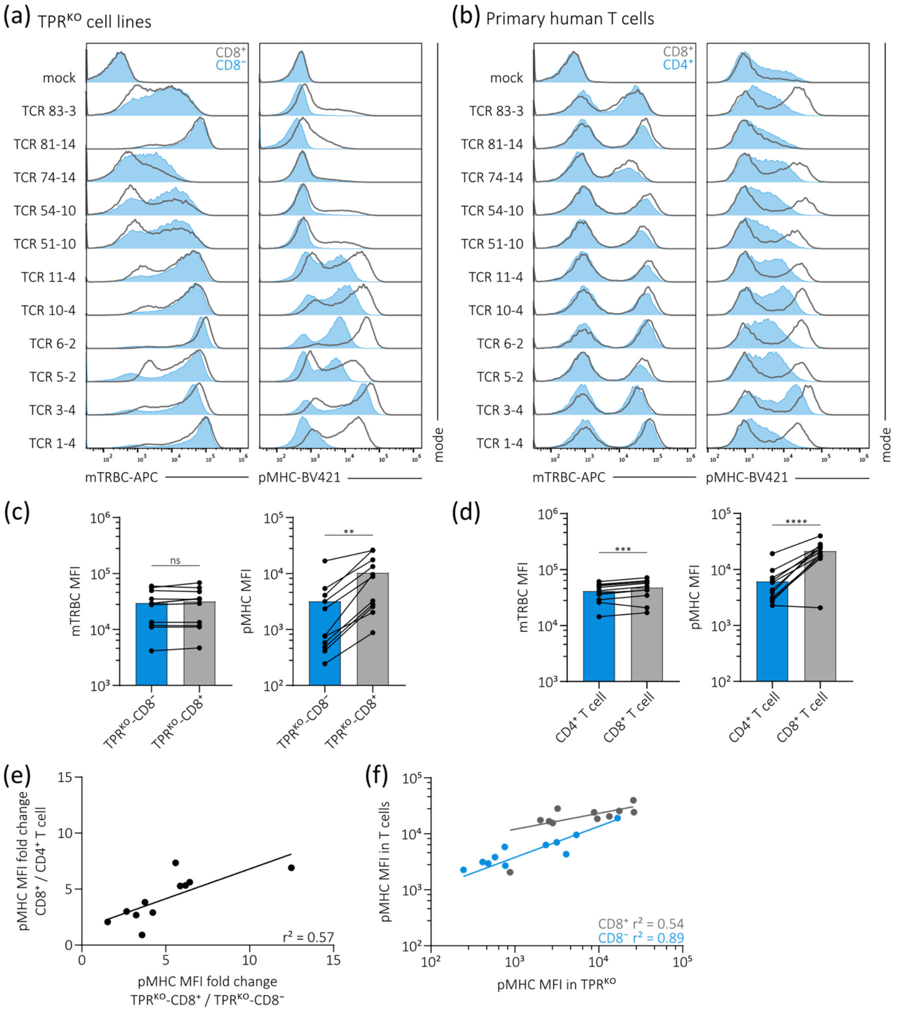

Suppl. Fig. 9. pMHC-multimer staining on TPR^{KO} cell lines is reliable and strongly correlates to primary T cells. (a/b) Histograms of mTRBC and pMHC-multimer staining of eleven A2/pp65-specific transgenically expressed TCRs in TPR^{KO} cell lines (a) and endogenous TCR-KO primary T cells (b). TPR^{KO}-CD8$^-$ and/or CD4$^+$ primary T cells in blue and TPR^{KO}-CD8$^+$ and/or CD8$^+$ primary T cells in grey. **(c/d)** Quantification of mTRBC and pMHC-multimer MFI in TPR^{KO} cell lines (c) and endogenous TCR-KO primary T cells (d). Each dot represents one of eleven individual TCRs. Statistical testing by two-tailed paired Student's t-test, ****P < 0.0001, ***P < 0.001, **P < 0.01. **(e)** Correlation of CD8αβ co-receptor dependency of pMHC-multimer staining between TPR^{KO} cell lines and primary T cells. Each dot represents one of eleven individual TCRs. Fitting by linear regression. **(f)** Correlation of pMHC-multimer staining between TPR^{KO} cell lines and endogenous TCR-KO primary T cells. Each dot represents one of eleven individual TCRs. Fitting by non-linear regression.
